# Unveiling the bidirectional link between electric vehicle sales and charging infrastructure: Evidence from 95 cities in China

**DOI:** 10.1016/j.isci.2024.111245

**Published:** 2024-10-24

**Authors:** Jianfeng Guo, Binbin Xu, Qi Cao, Siyao Liu, Fu Gu, Xuemei Zhang

**Affiliations:** 1 Institutes of Science and Development, Chinese Academy of Sciences, Beijing 100190, China; 2 School of Public Policy and Management, University of Chinese Academy of Sciences, Beijing 100190, China; 3 School of Economics & Management, Nanjing University of Science and Technology, Nanjing 210094, China; 4 Center of Engineering Management, Polytechnic Institute, Zhejiang University, Hangzhou 310015, China; 5 Department of Industrial and System Engineering, Zhejiang University, Hangzhou 310027, China; 6 National Institute of Innovation Management, Zhejiang University, Hangzhou 310027, China; 7 College of Humanities and Social Sciences, Nanjing University of Aeronautics and Astronautics, Nanjing 211521, China

**Keywords:** Energy sustainability, Social sciences, Economics

## Abstract

Constructing electric vehicle charging piles (EVCPs) is crucial for promoting electric vehicle (EV) sales. Yet, empirical evidence on the bidirectional relationship between EV sales and public EVCPs is limited, with most related works relying on simulations. We empirically investigate this relationship using panel vector autoregression (PVAR) across 95 Chinese cities from 2018 to 2022. Results show EVCPs significantly boost EV sales, especially in colder regions. Higher air pollution and purchase subsidies inhibit the promoting impact of EV sales on EVCPs. The propelling effect of EVCPs on EV sales is impaired in higher housing prices regions. A significant and positive bidirectional relationship exists between EVCPs and battery electric vehicles (BEVs) sales exists, but not with plug-in hybrid electric vehicles (PHEVs) present. Our findings have implications for stakeholders, such as the construction of fast charging infrastructure under lower temperatures, and differentiated charging demands of EVs with different powertrains need to be addressed.

## Highlights

• The bidirectional relationship between EVs and EVCPs is significantly positive
• Low temperature and low housing price significantly promote the impact of EVCPs on EVs
• High pollution significantly weakens the effect of EVs on EVCPs
• The bidirectional relationship only exists in EVCPs and BEVs

## Graphical abstract

**Figure:**
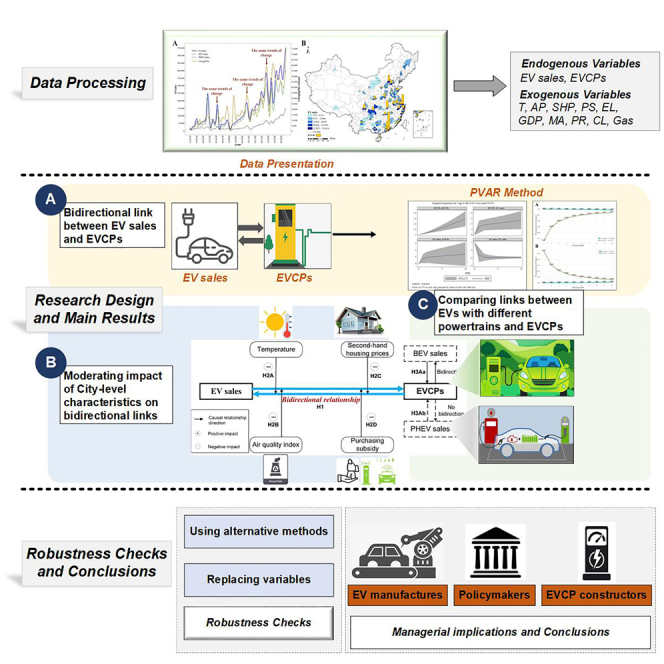

## Introduction

To mitigate climate change, promoting electric vehicles (EVs) adoption is an environmental priority of global societies. One of the major measures to achieve this objective is to construct public electric vehicle charging piles (EVCPs).^1^ Globally, there are about 3.78 million public EVCPs in 2023.^2^ Also, increasing EV adoption is accompanied by increased charging requirements, thereby demanding more public EVCPs. Therefore, theoretically, there exists a bidirectional link between EV sales and public EVCPs. Examining such a relationship would provide both theoretical and practical significance to stakeholders like policymakers, EV manufacturers, and EVCP constructors.

Currently, the extant literature primarily focused on identifying the unidirectional promotion effect of such charging infrastructure on the sales of EVs,^3^^,^^4^^,^^5^^,^^6^^,^^7^ while the impact of increasing EV ownership on the construction of EVCPs remains largely unknown. Admittedly, there were a few publications investigate this “chicken and egg” problem, but they suffered a series of flaws, which are summarized as follows. First, most of these studies analyzed the interdependence between EVs and EVCPs in term of an indirect network effect, in which either long-term market relationships or exogenous shocks induced by city-level characteristics are not included.^8^^,^^9^ Second, the present empirical evidence for the bidirectional relationship is limited, as most studies were based on mathematical modeling.^9^^,^^10^ Third, the only existing empirical studies, like Ma and Fan (2020) and Kalthaus and Sun (2021), used provincial or yearly data, ignoring monthly variations and city-level characteristics. In this work, we employ a more detailed city-level monthly panel dataset. Fourth, with global EV stock reached 40.5 million by the end of 2023,^2^ while there were only 10.1 million EVs in the year of 2020, when these empirical works had been conducted.^11^ This rapid growth likely alters the dynamics between EVs and EVCPs, calling for a closer, up-to-the-date empirical examination.

In this article, we pioneer empirically investigating the presence of the bidirectional relationship between EV sales and EVCPs based on a unique dataset that covers 95 cities in China, spanning from January 2018 to December 2022. We select China as the research context for two reasons. First, China tops global EV sales for consecutive 8 years. In 2023, the number of EV registrations in China reached 8.1 million, increasing by 35% relative to 2022, accounting for around 60% of the global share.^2^ Second, globally, China also ranks first in public EVCPs construction for 8 consecutive years and has 2.73 million public EVCPs by the end of 2023.^12^ Such a vast and representative market provides an ideal context for our empirical examination. Due to data availability, we include 95 Chinese cities, the list of which is given in Table S1. These cities’ EV sales and EVCPs cover 80% and 90% of the national figures, respectively, demonstrating sufficient representativeness.

Here, we consider the impact of four relevant city-level factors, i.e., temperature, air pollution, housing price, and purchase subsidies, on the bidirectional relationship between EVCPs and EVs. First, we introduce average temperature, which might affect battery performance.^13^^,^^14^ Second, the value of air quality index denotes the level of air pollution,^15^ as the indicator comprehensively reflects the concentrations of common air pollutants, including SO_2_, NO_2_, PM_2.5_, CO, PM_10_, and O_3_.^16^ Besides, present studies also explored both direct and indirect impacts of AQI on EV adoption.^15^^,^^17^^,^^18^ EV adoption could also subserve the reduction of PM_2.5_,^19^ one of the composition of pollutants of AQI.^16^ Therefore, we employ the AQI values as the indicator of air pollution. Third, we consider housing price since the previous literature shows that housing price is positively related to the purchase power of residents^20^^,^^21^ and the cost of EVCP construction.^22^ Fourth, we introduce purchase subsidies, as it is effective in incentivizing EV sales.^23^^,^^24^^,^^25^ In addition, we also extend this examination to the two types of EVs with different powertrains, i.e., battery electric vehicles (BEVs) and plug-in hybrid electric vehicles (PHEVs), which might lead to differentiated charging requirements.^26^ All the full names of abbreviations are shown in Table S2.

Methodologically, we employ a panel vector autoregression (PVAR) framework to examine the bidirectional link between EV sales and EVCPs, and to address potential endogeneity issues.^27^ By integrating panel data analysis and vector autoregression, the PVAR model provides a more robust analysis than Ordinary Least Squares (OLS),^27^ and has been extensively applied to explore the relationships between the selected variables.^28^^,^^29^^,^^30^ The PVAR framework consists of the following five parts. First, the panel unit root test is used to examine data stability. Second, the lag order is selected to obtain more appropriate model fitting. Third, the Granger causality test is rendered to apprehend the influencing mechanism between the EV sales and the public EVCPs. Fourth, using the generalized method of moments (GMM) to estimate the quantitative relationships between the selected variables. Fifth, impulse-response function and variance decomposition analysis are applied to examine long-short term relationships. Furthermore, to estimate the moderating effect of four city-level factors, we employ Two-stage least squares method (2SLS) with introducing two instrumental variables to address the potential endogeneity within the explanatory variables.^31^^,^^32^ For impact of the accessibility of public EVCPs on EV sales, we introduce the site area of commercial facilities (e.g., grocery stores and supermarkets^8^) as the instrumental variable for the endogenous public EVCPs. To evaluate the impact of EV stock on the deployment of EVCPs, we use gasoline prices as the instrumental variable for the endogenous cumulative EV sales. FMOLS and DOLS are also employed to enhance the reliability of the estimates in the presence of endogenous relationships. FMOLS adopts a semi-parametric approach to estimate the long-run parameters, and DOLS includes the leads and lags of the regressor to eliminate endogeneity, thus providing consistent estimators for long-term parameters. Additionally, we use the Granger causality test to further explore the bidirectional relationship between the sales of two types of EVs with different powertrains and public EVCPs.

This study contributes to the literature in the following three aspects. First, based on a unique city-level dataset, we extend the literature by exploring the cause-and-effect mechanism between the sales of electric vehicles and the public charging piles. This “chicken and egg” problem has drawn extensive attention from academia. The present studies modeled the interactions between EVCP investors and EV consumers based on game theories.^8^^,^^9^^,^^10^^,^^33^ Despite a series of insightful implications have been excavated via modeling and simulations, available empirical evidence is in extreme scarcity. Moreover, the endogenous issue within the linkage between EV sales and EVCPs might further compromise the validity of the current empirical observations^34^; This endogenous issue triggered by a reverse-causality relationship widely exists in the empirical studies on EVs and EVCPs.^8^^,^^9^^,^^35^ In this article, we employ an instrumental variable strategy to address the potential endogeneity.

Second, our study supplements the literature via unveiling the exogenous shock induced by city-level characteristics on EV sales and the accessibility of the public EVCPs, since no regional investigation on this bidirectional relationship has yet rendered. Targeted interventions and incentives are supposed to be specifically designed to examine the unique city-level characteristics. The effectiveness of EV promotion policies is under the influence of local characteristics, as evidenced by the former study.^36^ For example, Shang et al. (2024) observed that purchase subsidies are more effective in cities with more economically developed cities.^37^ This study only explains the impact of exogenous policy on EV promotion,^37^ while the nexus of the two sides of markets, i.e., EVs and public EVCPs, has not been investigated. Our study extends the EV sales literature by uncovering the impacts of city-level characteristics on the bidirectional link between EV sales and EVCPs. Specifically, here we show that the promotion effect of EVCPs on EV sales can be enhanced by lower temperature and housing price, while higher air pollution and purchase subsidies tend to weaken the propelling effect of EV sales on EVCPs. These observations shed light on the discussion of influential factors of charging infrastructure construction.

Third, our work further contributes to the rich literature on the diffusion of EVs with different powertrains, in this case, BEVs and PHEVs, propelling the understanding of differentiated charging requirements related to these heterogeneous power systems. The prior study focused on the need for tailored EVCPs for BEVs and PHEVs, but they always used national datasets, ignoring the impact of regional patterns on the linkage between EV sales and EVCP construction.^38^ We extend the literature by exploring the bidirectional causal relationship of EVCPs with EVs of different powertrains with the consideration of regional characteristics. Based on our pilot empirical examination, we excavate novel managerial insights that facilitate relevant decisions like proposing EV promotion strategies and deploying EVCPs.

## Results

### Evolution of China’s EV sales and EVCPs

**Figure 1:**
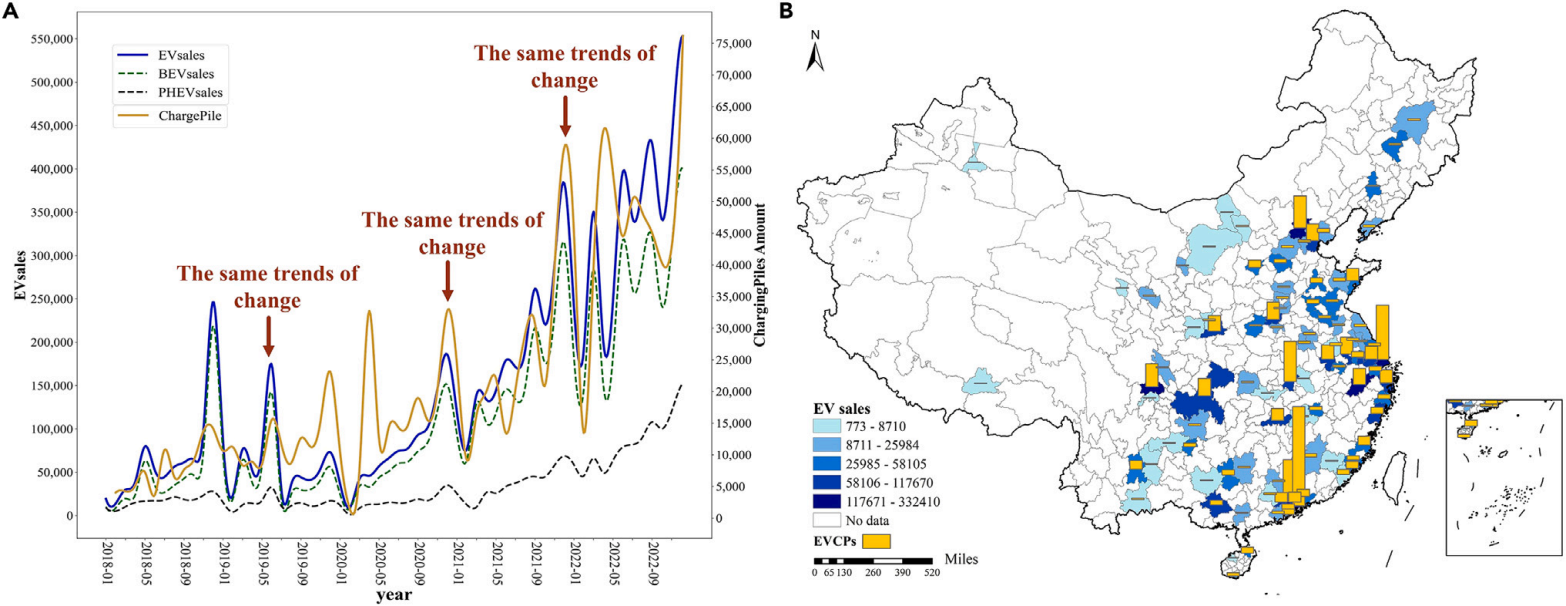
The trends and distributions of EV sales and EVCPs from 2018 to 2022 (A) The monthly trend of EV sales and public EVCPs from 2018 to 2022. (B) The distribution of EV sales and public EVCPs by the end of 2022. EV sales are indicated by the color in different regions, with higher colors intensity representing higher sales. The bar chart above each city in the map indicates the number of public EVCPs, with a higher column indicating more public charging piles.

Figure 1A illustrates the monthly trend of EV sales and EVCPs constructed from 2018 to 2022. A significant increase can be found in EV sales, around December each year. EV sales in summer (i.e., July, August, and September) also rise dramatically, implying that higher temperature may cause more automobile travel demand. This figure suggests a close relationship between EVs and EVCPs, which could be a bidirectional nexus. Figure 1B indicates that EVCPs are concentrated in the coastal rather than inland areas, which also have substantial EV sales. These observations suggest that the purchase preferences of EV owners might be related to their living environments.^7^

### The bidirectional relationship between EV sales and EVCPs

**Figure 2:**
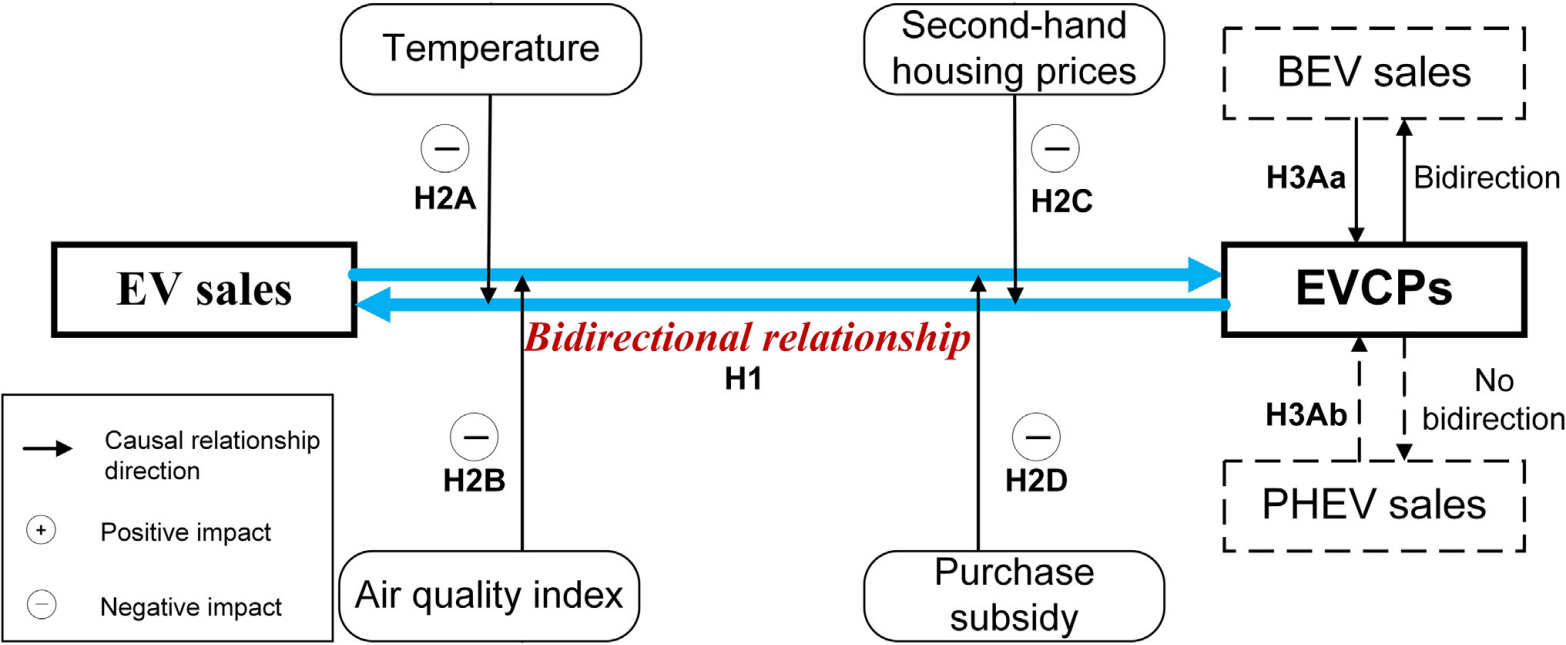
Model path hypothesis

Figure 2 illustrates the relations between the proposed hypotheses. The present studies demonstrated that the growth trend of EV sales and EVCPs have strong temporal and geographic couplings.^9^^,^^39^ For instance, accessibility of charging infrastructure remains a primary determinant of EV adoption, as outlined in consumers’ charging behavior.^40^ However, the empirical evidence of how EV sales affect public EVCPs is limited, as the former literature focused on the characteristics of EV-EVCP market.^8^^,^^9^ EVCP investors face a classic “chicken-and-egg” problem, where investment in charging infrastructure is disincentivized if EV sales volumes are insufficient to guarantee return on investment.^9^ Caillaud and Jullien (2003) pointed out that in the market’s two sides, one side always waits for the action from the other side following a game mechanism.^41^ Liu et al. (2024) further analyzed the interactive strategic decisions of participants in the EV-EVCP market using game, highlighting that EV adoption is influenced by charging accessibility. Additionally, EVCP investments are also contingent on the stock of EVs in the market. Thus, we propose.

Hypothesis (H1): *A significant positive bidirectional link exists between EV sales and EVCPs*.

Table 1 shows the results of the Granger causality test after examining data stationery and demonstrates a significant bidirectional causality between EV sales and EVCPs. Therefore, H1 is proved. The significance of the *p*-values (*p* = 0.013, 0, respectively) indicates that EVCPs are the Granger causes of EV sales; their values significantly affect the sales volumes of EVs. This observation aligns with a series of relevant EV publications.^42^^,^^43^ Furthermore, EV sales are also considered to be the Granger causes of EVCPs, as its *p*-values (*p* = 0.019, and 0, respectively) suggest that it significantly and positively affect EVCPs.

**Table 1:** Results of Granger causality test

Variables	Test items	*chi*^*2*^	*p*-values
*EV sales*	*EVCP*	6.236	0.013
*EVCPs*	*EV sales*	5.489	0.019

Panel 1 of Table S4 reveals that the values of EVCPs exhibit non-stationarity, thereby a first-order differencing transformation is applied. After the transformation, all the variables exhibit significant *p*-values, indicating the data becomes stationary. The cointegration tests confirm the existence of a ‘long-run equilibrium relationship’ among the selected variables, see Panel 2 of Table S4. Based on these findings, a PVAR model can be validly established. The lag order selection analysis suggests that using a lag order of 1 is appropriate for our study, see Panel 3 of Table S4.

The GMM estimation results shown in Table 2 suggest that positive relationship between EV sales and EVCPs as their *p*-values pass the 10% significance level. This further proves the existence of a significant bidirectional relationship between EV sales and EVCPs (H1); EV sales of lag one period (one year) increase by 1%, EVCPs increase by 0.078%, and EVCPs of lag one period increase by 1%, the EV sales are correspondingly increased by 0.145%. This observation is consistent with the findings of the previous studies^8^^,^^40^; The prior study also found the significant interdependence between EV adoption and the accessibility of the charging infrastructure.^8^

**Table 2:** Results of GMM estimation

*Variables*	*EV sales*	*EVCPs*
L1.*EV sales*	0.182	0.078∗∗
–	(0.803)	(2.343)
L1.*EVCPs*	0.145∗∗	1.036∗∗∗
–	(2.497)	(141.379)
*T*	−166.512∗∗∗	8.324∗
–	(-4.061)	(1.885)
*AP*	−84.834∗∗∗	5.998∗∗
–	(-4.597)	(2.644)
*SHP*	0.465	−0.001
–	(0.664)	(-0.020)
*PS*	−178.601∗∗	11.026
–	(-2.579)	(0.799)
*EL*	18,619.642∗∗	−3,520.149∗∗
–	(2.207)	(-2.120)
*GDP*	−1.321∗∗	0.084
–	(-2.241)	(1.340)
*MA*	−19.910∗∗	0.565
–	(-3.236)	(0.957)
*PR*	10,806.396∗∗	−857.364
–	(-2.392)	(-1.377)

∗, ∗∗, and ∗∗∗ indicate statistical significance at the 10%, 5%, and 1% level, respectively. The *t* statistics are reported in parentheses.

**Figure 3:**
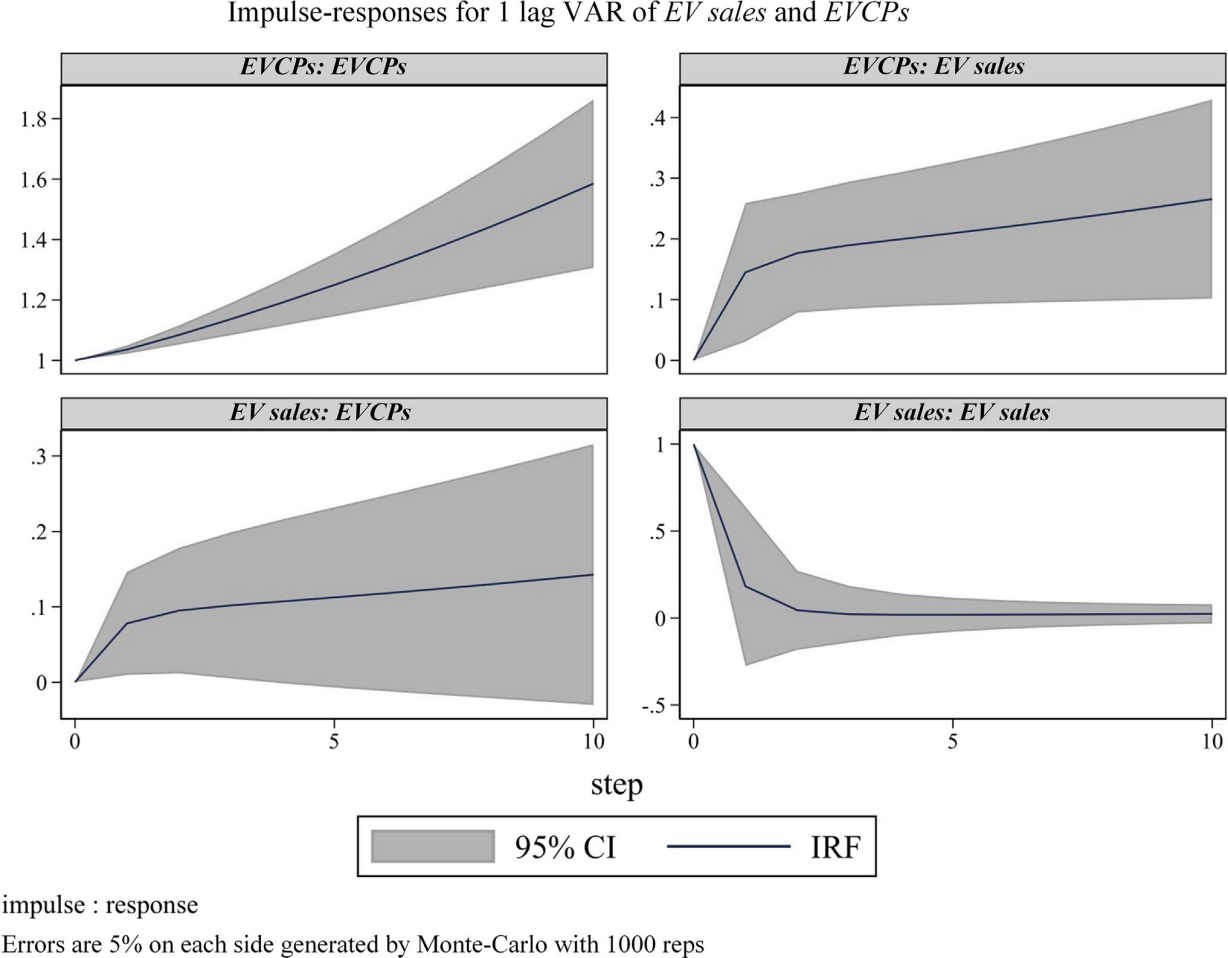
Variables impulse response The dash area indicates a 5% error in upper and lower bounds. Errors are 5% on each side generated by Monte-Carlo with 1000 reps.

As shown in Figure 3, to illustrate the relationships between EV sales and EVCPs, impulse response functions of the PVAR model are simulated. The periods are reflected on the horizontal axis. The first row of Figure 3 indicates that an increase in EVCPs leads to a sharp rise in EVCPs and a positive effect on EV sales. This effect exists in long-term of EV sales. The second row of Figure 3 indicates that the reaction of EV sales to shock in EVCPs always maintains a positive response state, eventually converging to a larger positive response value. The initial proliferation of EV sales has a positive impact on EV sales, but this impact gradually declines afterward.

**Figure 4:**
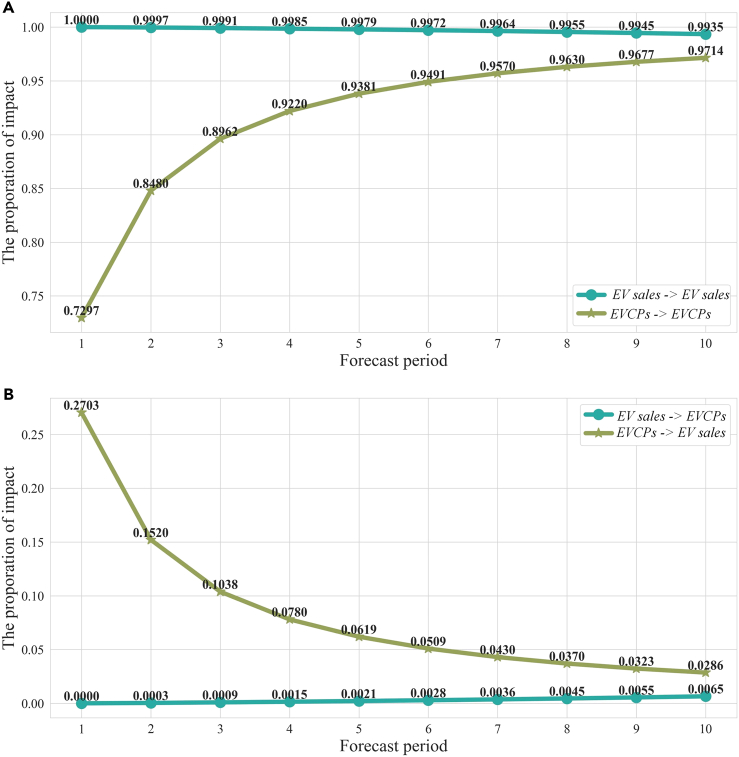
Variance decomposition analysis (A) The long-term impact of variables on itself. “EV sales -> EV sales” indicates the part of long-term influence of EV sales comes from itself. (B) The long-term impact of variables on another endogenous variable. “EVCPs -> EV sales” indicates the part of long-term influence comes from EV sales.

Figure 4 and Table S5 shows the variance of the decomposition of the selected variables. EV sales significantly promote the public EVCPs, gradually decreasing after the third period, see Figure 4A. According to Figure 4B, the change in EV sales is mainly self-driven and the impact gradually declines afterward. Figure 4 shows that there exists a short-term promoting impact of EV sales on EVCPs, reflecting a phenomenon that EVCP investors are highly responsive to the EV market. The self-driven EV sales imply that the impact of network externalities may be significant in EV market,^44^^,^^45^ that is, the value of a unit of product increases with the number of unit sold.^9^

### Impact of city-level characteristics on the bidirectional relationship

**Figure 5:**
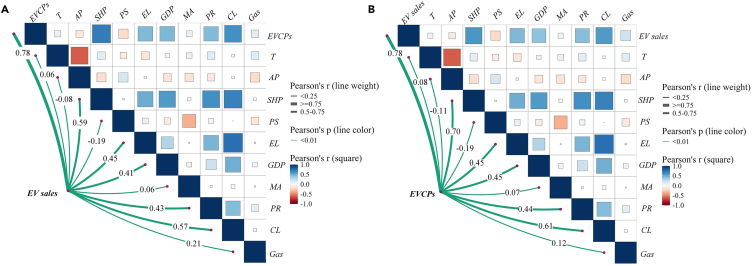
Correlation analysis of variables The four endogenous variables are represented as nodes that connect the heatmap matrix. The thickness of the connecting lines depends on the degree of correlation. The green colored line represents significance at the 99% level. The heatmap matrix displays the degree of correlation between other variables, with darker colors indicating a stronger correlation. (A) The correlation between EV sales and other variables. (B) The correlation between EVCPs and other variables.

This Subsector demonstrates the impact mechanisms of four exogenous factors, including temperature, air pollution, housing price, and purchase subsidies, on the mutual causal relationship between EVCPs and EV sales are discussed. Considering the endogenous issue, using 2SLS method, two instrumental variables (IV) are applied including gasoline price and commercial land price. An appropriate IV should be able to explain the independent variable and not directly affect dependent variable. Gasoline price has been found to have a significant impact on EV sales.^46^ High utilization cost of conventional fuel vehicle might be one of the reasons forcing consumers to consider EVs.^47^ Additionally, Li et al. (2017) also used gasoline price as the instrumental variable to explore the causal relationship of EV sales on EVCP. For the public EVCPs, which usually are deployed at workplace parking lots, shopping centers, grocery stores, restaurants, dealers, and gasoline stations.^8^ These areas commonly have the nature of commercial service, with deploying EVCPs for attracting customers for their main business. Thus, we use commercial land use as the instrument variable for the public EVCPs. The Pearson correlation between all the included variables in Figure 5.

#### Influence of temperature

Temperature significantly influences the propensity of EV sales and EVCP deployment,^48^^,^^49^^,^^50^ since the factor affects the operation and charging performance of EV batteries.^48^ Hao et al. (2020) found that in winter, charging frequency is 24% higher than in other seasons although there are range limitations caused by colder weather.^51^ Yuksel and Michalek (2015) show that the capacities of EV batteries would be diminished rapidly in lower temperature, leading to substantial reductions in EV range.^52^ Besides, Gao et al. (2023) showed that colder weather poses a greater demand on charging, with this regard, providing better accessibility to charging infrastructure can promote EV adoption. Therefore, it is reasonable to assume that under lower temperatures, the EV sales could be motivated by increased public EVCPs, due to increased charging demands. However, there still exists controversy regarding the influence of temperature on EV sales. For instance, based on the seasonal sales statistics, Haidar and Rojas (2022) showed that lower temperature strengthens the promotion effect of EVCPs on EV sales, while Hamwi et al. (2022) reached the opposite conclusion.^53^ Hence, here we propose.

Hypothesis (H2A): *Lower temperature enhances the impact of EVCPs on EV sales*.

Table 3 show that temperature can significantly weaken the promoting effect of the public EVCPs on EV sales as the *p*-values are all significant, proving the validity of H2A. When the temperature declines by 1%, the promoting impact of the public EVCPs on EV sales would be increased by 0.004%. A reasonable explanation is that the reduction of EV range is perceived in an extremely cold environment.^48^ Hence, building charging piles in low temperature periods or regions could notably propel EV sales. This observation is in line with Liu and Wu (2023).^54^

**Table 3:** Estimation of the moderating effect of temperature

Variables	*EV sales*	*EVCPs*
	(1)	(2)
*EVCPs*	0.270∗∗∗	–
–	(4.640)	–
*EVCPs × T*	−0.004∗∗	–
–	(-2.400)	–
*EV sales*	–	0.898
–	–	(0.953)
*EV sales × T*	–	0.040
–	–	(1.234)
*T*	24.518∗	−1.607
–	(1.861)	(-0.537)
*AP*	1.743	−9.689
–	(0.808)	(-0.997)
*SHP*	0.153∗∗∗	−86.997
–	(3.987)	(-1.255)
*PS*	94.829∗∗∗	−556.890∗∗∗
–	(3.364)	(-7.552)
*EL*	634.531∗∗	0.817∗∗∗
–	(2.039)	(4.886)
*GDP*	0.082∗∗∗	4,987.476∗∗∗
–	(3.725)	(4.716)
*MA*	−2.780∗∗∗	−0.081
–	(-4.162)	(-0.994)
*PR*	−178.631	552.923
–	(-0.384)	(0.375)
*First-stage IV*	87.803∗∗∗	−178.668∗∗∗
–	(7.870)	(-4.260)
*Wald chi*^*2*^	8,812.270	11,724.860

This table reports the results of the moderating effects of temperature. For 2SLS estimation, the *t*-value are reported in the first-stage, and the *z*-values are reported in the second stage. ∗, ∗∗, and ∗∗∗ indicate statistical significance at the 10%, 5%, and 1% level, respectively.

#### Influence of air pollution

Air pollution influences EV sales via stimulating public environmental awareness,^26^^,^^55^ while whether this stimulating effect could be replicated on EV sales to EVCPs is not excavated. First, notably, health concerns significantly affect the decisions to purchase behaviors,^3^ since the exposure to air pollution would cause both physical and mental problems, for example, human respiratory diseases and compromised work efficiency.^56^^,^^57^ Zhao et al. (2024) demonstrated that air pollution has a direct impact on EV adoption^17^ due to consumers’ concerns over health burdens related to air pollution. Comparatively, the impact of air pollution on EVCPs is rarely studied, although EV sales could stimulate increased demand for EVCPs. Second, the use of EVs might be confined in highly polluted areas where fossil fuels are used for power generation, since charging infrastructure magnifies the environmental performance of electricity, which are directly related to the environmental benefits of EVs.^58^ It is well known that the environmental performance of transportation electrification is linked to the sources of electricity.^59^ In the regions where a substantial proportion of electricity is derived from renewable sources (e.g., wind, solar, and hydroelectric power), EVCPs would amplify the environmental advantages of these energies via facilitating EV usage, while in the region where fossil fuels (e.g., steam coal and crude oil) are used as the energy sources, increased EVCPs could aggravate anabatic pollution.^60^^,^^61^ Since China currently uses steam coal as the primary energy source of the national power grid,^62^ the expected environmental benefits of EVs would be compromised, thereby the use of EVs might be confined, particularly in highly polluted areas. In this regard, the requirement for charging infrastructure in such areas would be diminished.^63^ Therefore, we speculate that in highly polluted areas, EVCP construction might be constrained, as the use of EVs cannot offer the anticipated environmental benefits.

Hypothesis (H2B): *Higher air pollution weakens the impact of EV sales on EVCPs*.

For air pollution, air pollution significantly positively moderates the promotion effect of EV sales on EVCPs as the *p*-values are all significant, see Table 4. Hence, H2B is validly proved, indicating that higher pollution negatively moderates the promotion effect of EV sales on the accessibility of the public EVCPs. One possible reason is due to the lack of environmental governance measures, thus hindering the construction of public EVCPs. In addition, the environmental performance EVCPs is highly dependent on the sources of electricity. In the regions that depend heavily on coal or oil for electricity generation, EVs may not exhibit sufficient environmental advantage over gasoline vehicles.^64^ This partially agrees with the observation of Zahoor et al. (2023), which suggested that air pollution is related to the lag behind of public EVCP construction.^65^ This observation provides an important implication that the deployment of the public EVCPs should be decided according to local environmental conditions.

**Table 4:** Estimation of the moderating effect of air pollution

Variables	*EV sales*	*EVCPs*
	(1)	(2)
*EVCPs*	0.187∗∗	–
–	(2.897)	–
*EVCPs × AP*	−0.0001	–
–	(-0.141)	–
*EV sales*	–	2.686∗∗∗
–	–	(3.386)
*EV sales × AP*	–	−0.021∗
–	–	(-1.824)
*T*	−4.854	−27.587∗
–	(-1.089)	(-1.702)
*AP*	−0.957	14.397
–	(-0.265)	(1.435)
*SHP*	0.157∗∗∗	0.733∗∗∗
–	(3.407)	(5.094)
*PS*	73.748∗∗∗	−497.142∗∗∗
–	(3.567)	(-7.853)
*EL*	722.522∗∗	4,690.139∗∗∗
–	(2.618)	(4.914)
*GDP*	0.079∗∗∗	−0.106
–	(3.435)	(-1.442)
*MA*	−3.080∗∗∗	−0.968
–	(-5.125)	(-0.467)
*PR*	−196.277	1,026.242
–	(-0.421)	(0.693)
*First-stage IV*	78.879∗∗∗	209.248∗∗∗
–	(6.770)	(7.420)
*Wald chi*^*2*^	9,542.140	11,276.280

This table reports the results of the moderating effects of air pollution. For 2SLS estimation, the *t*-value are reported in the first-stage, and the *z*-values are reported in the second stage. ∗, ∗∗, and ∗∗∗ indicate statistical significance at the 10%, 5%, and 1% level, respectively.

#### Influence of housing price

Housing prices affect the prices of various products and services, including commodity prices, labor expenses, and social services.^9^ Housing prices would also be added the costs of EVCP construction.^40^ This hinders the construction of public charging infrastructures due to increased land costs.^66^ lower housing prices could incentivize investors to expand the development of such charging infrastructure. Consequently, such infrastructures have been frequently constructed in remote areas where housing price is low.^40^ Increased accessibility to charging infrastructure stimulates the adoption of EVs.^47^ However, in the regions with higher housing prices, the costs of installing EVCPs are also higher, thereby reducing the accessibility to charging infrastructure. Consequently, higher housing prices inhibit EV adoption. In addition, in the regions with higher housing prices, residents tend to have higher incomes and to be less price sensitive.^9^ Due to the lower average usage costs of EVs, these high income groups are less likely focus on this advantage, while other factors such as parking lot availability,^67^ manipulation and comfort perception^25^ can significantly limit EV adoption. Accordingly, we propose.

Hypothesis (H2C): *Higher housing price weakens the bidirectional relationship between EVCPs and EV sales*.

Table 5 shows that the locations with higher housing price would weaken the promotion effect of the public EVCPs on the EV sales to a certain extent, as the *p*-values are significant. Hence, H2C can be proved. However, this weaken impact is comparatively small. This result consistence with reality because the prices of second-hand housing are mainly determined by two components, i.e., land price and construction cost.^68^ The high housing price is commonly accompanied by high land prices in surrounding areas.^69^ Subsequently, the construction of public EVCPs in high-priced areas would incur more substantial costs.^40^

**Table 5:** Estimation of the moderating effect of housing price

Variables	*EV sales*	*EVCPs*
	(1)	(2)
*EVCPs*	0.302∗∗∗	–
–	(4.744)	–
*EVCPs × SHP*	−2.71E-06∗∗	–
–	(-2.800)	–
*EV sales*	–	6.618
–	–	(1.232)
*EV sales × SHP*	–	−0.000
–	–	(-0.870)
*T*	−5.406	15.439
–	(-1.360)	(0.521)
*AP*	−0.796	6.706
–	(-0.491)	(0.617)
*SHP*	0.332∗∗∗	1.077∗∗
–	(9.278)	(2.277)
*PS*	86.976∗∗∗	−389.717∗∗
–	(3.517)	(-2.343)
*EL*	544.571∗	1,295.673
–	(1.693)	(0.362)
*GDP*	0.092∗∗∗	−0.246
–	(4.264)	(-1.392)
*MA*	−2.712∗∗∗	9.225
–	(-4.164)	(0.903)
*PR*	−453.547	−180.336
–	(-0.985)	(-0.075)
*First-stage IV*	77.643∗∗∗	47.107
–	(9.840)	(1.470)
*Wald chi*^*2*^	9,542.150	5,310.030

This table reports the results of the moderating effects of housing price. For 2SLS estimation, the *t*-value are reported in the first-stage, and the *z*-values are reported in the second stage. ∗, ∗∗, and ∗∗∗ indicate statistical significance at the 10%, 5%, and 1% level, respectively.

#### Influence of purchase subsidies

Purchase subsidies are an effective instrument to increase EV sales.^70^^,^^71^ However, high purchase subsidies occupy the relevant budget for the promotion of electric vehicles, resulting in the lagging construction of supporting facilities like EVCPs.^72^ Besides, lower subsidies make the public pay more attention to the performance of EVs. Therefore, consumers purchase EVs in a more reasonable manner, and use EVs more frequently, further stimulating EVCP construction.^25^ Subsidies on the EV prices, integration with other policies, including EVCP construction subsidies and operation subsidies,^9^ all need for specific policy designs, to maximize the expansion and efficacy of EVs and charging infrastructure. EV purchase subsidies are proved to be less effective in EV adoption compared to alternative policies such as EVCP construction subsidies and operational grants.^73^ This underscores the necessity of tailoring policies to facilitate EV adoption and EVCP construction. Additionally, the literature points out that the propelling effect of purchase subsidies is decreased with the EV sales, demonstrating a diminishing marginal effect.^37^ In this regard, higher purchase subsidies might be less motive for EVCP construction. Yet, to the best of our knowledge, the moderating impact of purchase subsidies between EV sales and EVCPs remains unknown. Hence, we propose the following hypothesis.

Hypothesis (H2D): *Lower purchase subsidies strengthen the bidirectional relationship between EV sales and EVCPs*.

Considering EV purchase subsidies, Table 6 indicate that increases in purchase subsidies would weaken the promotion effect of EV sales on EVCPs, as the *p*-values are both significant. This confirms the validity of H2D. One potential reason can be that substantial EV purchase subsidies largely consume the governmental budget for transportation electrification, resulting in the lagging construction of supporting facilities like public EVCPs.^72^ Another possible underlying reason is that lower purchase subsidies may reduce fraudulent conducts that aim at acquiring such subsidies. With reduced occurrences of frauds, EVs are more likely to be purchased for usage, and consequently the demand and construction of the public EVCPs are noticeably promoted. This argument may further lead to the emphasis on the characteristics of EVs, as the previous literature suggests that EVs’ attributes such as comfort, manipulation, and space could largely affect their sales volumes.^25^^,^^74^

**Table 6:** Estimation of the moderating effect of purchase subsidies

Variables	*EV sales*	*EVCPs*
	(1)	(2)
*EVCPs*	0.168∗∗∗	–
–	(5.552)	–
*EVCPs × PS*	0.011	–
–	(1.362)	–
*EV sales*	–	2.468∗∗
–	–	(2.899)
*EV sales × PS*	–	−0.393∗∗
–	–	(-3.228)
*T*	−4.950	−10.661
–	(-1.206)	(-0.884)
*AP*	−1.475	−2.053
–	(-0.905)	(-0.438)
*SHP*	0.171∗∗∗	0.570∗
–	(3.911)	(1.713)
*PS*	43.752	46.136
–	(1.130)	(0.232)
*EL*	779.379∗∗	3,285.507∗
–	(2.509)	(1.650)
*GDP*	0.074∗∗∗	0.176∗∗
–	(3.377)	(2.526)
*MA*	−3.006∗∗∗	−0.978
–	(-4.929)	(-0.315)
*PR*	−194.225	922.701
–	(-0.423)	(0.699)
*First-stage IV*	166.891∗∗∗	174.967∗∗∗
–	(9.740)	(3.660)
*Wald chi*^*2*^	8,761.170	12,965.060

This table reports the results of the moderating effects of purchase subsidies. For 2SLS estimation, the *t*-value are reported in the first-stage, and the *z*-values are reported in the second stage. ∗, ∗∗, and ∗∗∗ indicate statistical significance at the 10%, 5%, and 1% level, respectively.

### Electric vehicle types

EVs of different powertrains, i.e., BEVs and PHEVs, could lead to differentiated charging requirements, as the former category solely depends on batteries while the latter category uses both fossil fuels and batteries.^26^^,^^75^ BEVs solely depends on their batteries, therefore exhibiting a greater dependency on EVCPs. The literature further confirms that the adoption of BEVs correlates with the accessibility of charging infrastructure.^76^ In contrast, PHEVs exhibit a comparatively lower reliance on charging infrastructure due to the possession of combustion engines. Present evidence confirmed that PHEV owners use charging facilities less frequently.^77^ This flexibility often reduces the urgency for widespread charging infrastructure in the areas predominantly served by PHEVs. Such a difference would not only affect the construction of EVCPs but also result in differentiated bidirectional relationships between the sales of EVs of different powertrains and EVCPs. In this regard, we propose the hypothesis.

Hypothesis (H3Aa): *A bidirectional relationship exists between BEV sales and EVCPs*.

Hypothesis (H3Ab): *A bidirectional relationship does not exist between PHEV sales and EVCPs*.

In Tables 7 and 8, the results indicate a significant bidirectional relationship between BEV sales and EVCPs, while it fails to achieve the same results for PHEVs. This demonstrates that the bidirectional relationship only exists between BEVs and EVCPs, confirming H3Aa and H3Ab. Specifically, the significance of the *p*-values (*p* = 0.014 and 0.048, respectively) in Table 7 indicates that public EVCPs impart a greater promoting effect on BEV sales. This piece of empirical evidence indicates that increasing BEV sales volumes also advances the deployment of public EVCPs, being compatible with the outcome of Zhang et al. (2022).^78^ However, for PHEV sales, the bidirectional relationship is invalid, as their *p*-values (*p* = 0.744 and 0.589, respectively) suggest that EVCPs are not the Granger cause of PHEVs, see Table 7. These observations demonstrate that BEVs are highly dependent on EVCPs due to the fact that this type of EVs is completely powered by electricity stored in their batteries, while PHEVs can also consume gasoline. The lag order criteria selected are presented in Table S6.

**Table 7:** Outcomes of the Granger causality test with different powertrains

Variables	Test items	*chi*^*2*^	*p*-values
***BEV* sales**			

*BEV*	*EVCPs*	5.996	**0.014**
*EVCPs*	*BEV*	3.915	**0.048**

***PHEV* sales**			

*PHEV*	*EVCPs*	0.107	**0.744**
*EVCPs*	*PHEV*	0.293	**0.589**

**Table 8:** Panel VAR estimation of BEV and PHEV

Variables	BEV	EVCPs	PHEV	EVCPs
L.*BEV*	−0.040	0.096∗∗	–	–
–	(-0.221)	(1.979)	–	–
L.*PHEV*	–	–	0.314	3.457
–	–	–	(0.441)	(0.541)
L.*EVCPs*	0.077∗∗	1.041∗∗∗	0.022	0.276
–	(2.449)	(139.292)	(0.327)	(0.465)
*T*	−146.943∗∗∗	6.447	−121.104∗∗∗	−857.226∗∗∗
–	(-3.562)	(1.347)	(-4.807)	(-4.165)
*AP*	−73.889∗∗∗	5.108∗∗	−7.998	−59.445
–	(-3.974)	(2.056)	(-0.639)	(-0.593)
*SHP*	0.944∗	−0.037	0.116	2.071
–	(1.694)	(-0.446)	(0.340)	(0.631)
*PS*	−75.873	2.740	−20.408	−253.113
–	(-1.097)	(0.172)	(-0.267)	(-0.365)
*EL*	15,975.357∗∗	−3,413.536∗∗	3,987.779	51,904.624
–	(2.535)	(-2.017)	(0.927)	(1.319)
*GDP*	−0.870∗	0.048	0.161	2.176
–	(-1.684)	(0.733)	(0.501)	(0.865)
*MA*	−17.593∗∗	0.363	−13.926∗∗∗	−43.092∗∗
–	(-2.998)	(0.570)	(-5.560)	(-2.280)
*PR*	6,969.491∗	−590.114	31,262.438∗∗∗	586,674.856∗∗∗
–	(1.757)	(-0.965)	(4.226)	−4.626

∗, ∗∗, and ∗∗∗ indicate statistical significance at the 10%, 5%, and 1% level, respectively. The *t* statistics are reported in parentheses.

### Robustness checks

In this Subsector, we conducted a series of strategies to test the robustness of the findings, including using alternative models (i.e., 2SLS, to address the potential endogenous bias) and rendering variable placements (the definitions, data sources and justifications of the substitute variables are shown in Table S7). We also apply the FMOLS and DOLS to confirm the validity and consistency of our results with also considering endogenous issues; these measures are widely used for robustness checks.^34^ Table S8 provides the outcomes of our robustness checks.

#### Robustness check of bidirectional relationship

The alternative model, i.e., 2SLS, is applied to examine the existence of the bidirectional relationship between EV sales and EVCPs. Panel 1 of Table S8 (the outcomes of 2SLS) shows that the *p*-values of EV sales and EVCPs are all significant, indicating that H1 is valid. The results of the GMM estimates in Table 2 are robust, that is, a significant bidirectional relationship between EV sales and EVCPs exists.

#### Robustness check of moderating effect of city-level characteristics

We use four substitute variables as the new measurements to check the robustness of our results with city-level characteristics. Variable replacements Panel 2–5 of Table S8 display the outcomes of city-level characteristics’ moderating impact on the bidirectional relationship. We also apply the FMOLS and DOLS as the new estimation to check these moderating impacts. Panel 2 of Table S8 support the validity of H2A; lower temperature has a significant enhancing impact on the propelling effect of the public EVCPs to EV sales volumes. Panel 3 of Table S8 proves the validity of both H2B, as air pollution inhibits the promotion effect of EV sales on the public EVCPs. Panel 4 of Table S8 does not support H2C; the prices of newly built houses seem have no significant negative impact on the promotion effect of EV sales to the public EVCPs. The reason might be the newly built housing price could not completely reflect the housing market due to government regulations.^79^^,^^80^ Panel 5 of Table S8 supports H2D, as substantial EV purchase subsidies hinder the promoting impact of EV sales on EVCPs.

#### Robustness check of the bidirectional relationships with different powertrains

We also use the public newly built EVCPs as the measurement of accessibility of the public EVCPs by employing Granger causality test. Panel 6 and 7 of Table S8 show that newly built EVCPs generally have a causal relationship with BEV sales but not with PHEV sales, thereby confirming the robustness of our findings by proving the validity of H3Aa and H3Ab.

## Discussion

### Remarks on the results

In comparing our research with the relevant literature, we have observed several intriguing differences. The first difference lies in our unique dataset that covers 95 cities in China; this work would provide more comprehensive evidence to support our conclusions, as the previous studies primarily use macroscopic datasets such as national data^46^ and provincial data.^40^ We explore the quantitative relationships between EV sales and the public EVCPs, compared to the prior study measuring EVCPs proxied by public financial expenditure,^36^ which would compromise the validity of their empirical results. The second difference is within the selected econometric method, i.e., the PVAR, which is particularly applicable in addressing potential endogeneity. This sets our study apart from the previous literature that employs panel OLS,^6^^,^^25^ GLM,^81^ and mixed-effects regression,^39^ where endogeneity issues persist. The third difference can be found in our observations. We pioneer to provide the empirical evidence that supports the existence of a bidirectional relationship between EV sales and public EVCPs, while most of the relevant studies based on mathematical modeling.^9^^,^^10^ In detail, our findings reveals that the EV market exhibits a transient promotional effect on EVCPs, mirroring investors’ swift responsiveness, offering empirical evidence to support the theoretical speculation of Levinson and West (2018). The detailed information of all variables is given in Table 9. The descriptive statistics are also summarized in Table 10.

**Table 9:** Variables, definitions, and data sources

Variables	Definition	Justification	Data source
**Endogenous variables**			

*EV sales*	The monthly sales volumes of electric vehicle.	This variable measures the level of EV adoption.^19^^,^^24^	China Banking and Insurance Regulatory Commission (CBIRC)
*EVCPs*	The number of monthly constructed public EVCPs.	This variable indicates the construction progress of public EVCPs in each included city.^39^	China Electric Vehicle Charging Infrastructure Promotion Alliance

**Exogenous variables**			

*T*	The monthly average temperature.	It can significantly affect the available battery power and driving range of EVs.^49^ The impedance variation is based on the fact that temperature is intrinsically linked to how quickly the electrochemical reaction occurs inside battery cells.^48^	ERA5-Land Dataset released by European Union and European Center for Medium-Range Weather Forecasts (ECMWF) (cds.climate.copernicus.eu/cdsapp#!/dataset/reanalysis-era5-land-monthly-means?tab=overview)
*AP*	Air pollution is reflected by each city’s air quality index (AQI) every month.	The higher pollution triggers environmental willingness to pay,^26^^,^^106^ thereby promoting the sales of environmentally friendly products like EVs.	PM2.5 history data (www.aqistudy.cn/historydata/).
*SHP*	Monthly averaging second-hand housing price.	Housing price is positively related to public purchase power^20^ and EVCP construction cost. Second-hand housing is more reflective of the housing market due to government regulations on newly-built houses.^79^^,^^80^	Anjuke’s official platform (hechi.anjuke.com/sale/?from=HomePage_TopBar) is a professional website providing monthly housing information for each city in China.^107^ The data from this platform is extensively used in related works.^108^^,^^109^
*PS*	The monthly purchase subsidies in each included city are promulgated by central and local governments in BEVs and PHEVs.	Refer to the prior study,^40^ we adopt the upper limit of the monthly EV subsidy due to the subsidy is divided according to driving range of EVs. We also add up the purchasing subsidy for BEVs and PHEVs.	The websites of related governments, including The Development and Reform Commission, the Department of Industry and Information Technology, the Department of Finance, the Department of Transport, and the Municipal Government Office.
*EL*	Education level in each city.	To reflect education level comprehensively, this factor is measured by using the following method which widely applied in former research.^110^^,^^111^^,^^112^ Education level = primary education level + secondary education level + higher education level, where primary education level = the proportion of illiterate and semi-illiterate population × 2 years + proportion of population with primary education × 6 years. The secondary education level = proportion of population with junior high education × 9 years + proportion of population with senior high education × 12 years. Higher education level = proportion of population with junior college education or higher education × 16 years.	Statistical yearbooks of each province and China population and employment statistical yearbook.
*GDP*	GDP per capita in each city.	GDP can serve as an indicator of a region’s economic development, the enhancement of consumer capacity, the innovation in social organization, the overall quality of life, and the efficiency of resource allocation and output generation.^113^^,^^114^^,^^115^^,^^116^ Therefore, GDP per capita is controlled in this paper with consideration of inconsistent population distribution. Since the original dataset of this parameters is presented annually, a differencing process^26^ is used to transform the data into a monthly form.	Statistical yearbooks of each province.
*MA*	The monthly average trading volume of each EV enterprise’ stock.	Market activity can be measured by trading volume.^117^ Average trading volume is estimated by multiplying daily shares traded times the average price of a traded share.^117^ Former scholar has explored the EV share is related to their market performance.^118^ The trading volume is an indicator that reflects the change in investors’ expectations for corporate performance, which affect the financing of this enterprise.^119^ Since financing is a key factor of the marketing of EVs and the construction of EVCPs,^120^ we include this variable in our assessment.	The financial terminal named Choice of EastMoney (https://www.eastmoney.com/) and TUSHARE Pro API (c)
*PR*	Purchasing restriction	Several cities of China have implemented the policy restriction of fuel vehicles with the consideration of traffic congestion and air pollution.^36^^,^^121^ Due to data availability, we use purchasing restriction controlling government intervention on EV industry.	Official website of each city and reference to Gao et al. (2023)^36^
*CL*	Commercial land use	The business runners of grocery stores and shopping malls build EVCPs near their business to attract customers for the main business.^9^ Li et al. (2017) also considers the impact of grocery stores and supermarkets on EVCPs.^8^ The larger commercial construction land, the more space for EVCP deployment.	Statistical yearbook of Chinese urban construction
*Gas*	Retail prices of 92# gasoline.	In line with the EVs literature,^47^^,^^122^ we employ the price of the No.92 gasoline, the most widely consumed fossil fuel in China.	The TUANYOU platform (www.tuanyou.net/youjia/) is a website that provides the historic gasoline prices in China.^123^

**Table 10:** Data statistics

Variables	N	Mean	Standard deviation	Min	Median	Max	Skewness	Kurtosis
*EV sales*	5,700	1,563.748	3,274.180	0	405.500	57,985.000	5.445	50.286
*BEV*	5,700	1,212.298	2,429.763	0	310.000	32,576.000	4.500	30.059
*PHEV*	5,700	351.407	1,013.922	0	61.000	25,409.000	9.370	149.322
*EVCPs*	5,700	5,912.405	13,542.972	0	1,782.500	207,577.000	6.194	55.110
*T*	5,700	15.703	9.675	−20.254	17.173	32.596	−0.687	−0.117
*AP*	5,700	60.056	27.074	14.458	53.540	206.927	1.455	2.880
*SHP*	5,700	13,680.622	11,179.001	4,025.000	10,426.500	86,429.000	2.976	9.796
*PS*	5,700	4.016	2.481	1.740	3.100	14.400	1.423	1.361
*EL*	5,700	1.842	2.218	0.153	1.061	12.716	3.241	11.249
*GDP*	5,700	7,386.795	3,468.598	484.244	6,695.202	43,654.109	0.998	2.809
*MA*	5,700	183.077	49.140	114.425	168.912	353.761	1.092	1.395
*PR*	5,700	0.109	0.312	0	0	1.000	2.502	4.262
*CL*	5,700	24.086	25.410	0.890	14.390	137.260	2.114	4.989
*Gas*	5,700	7.143	0.852	5.370	7.045	10.305	0.280	0.172

To the best of our knowledge, the previous studies rarely discuss the potential interaction between EV sales and EVCPs from a city-level perspective. In this paper, we fulfill this knowledge gap by including four city-level characteristics, i.e., temperature, air pollution, housing price, and purchase subsidies, in the examination of the bidirectional relationship between EVCPs and EVs. For temperature, it acts as a moderator, that reduces the stimulatory effect of EVCPs on EV sales. This suggests that constructing EVCPs in low temperature periods or regions could propel EV sales. Air pollution negatively moderates the relationship between EV sales and EVCPs, and such an inhibition effect can be originated from non-environmentally friendly sources of power generation. The extant literature mainly examined the promotion effect of high economic status on EV adoption,^47^^,^^82^ while little attention has been paid on the hindrance effect of high housing price on the EV adoption. Our work supplements the empirical evidence that confirms the presence of such a hindrance effect. Furthermore, we also underscore the negative impact of high EV purchase subsidies on the accessibility of public EVCPs. Here we provide side evidence to demonstrate the existence of a diminishing marginal effect, as well as to highlight the significance of EVs’ performance.

In addition, we empirically investigate the charging requirements of the two EVs of different powertrains, i.e., BEVs and PHEVs. Our findings suggest that a significant bidirectional relationship between BEV sales and public EVCPs presents, while the bidirectional relationship between BEV sales and public EVCPs is insignificant. This can be attributed to their differentiated powertrains, as BEVs are totally relied on electricity. Our empirical observation is compatible with the sales statistics of these types of EVs (Figure 1A), lower temperature accelerates the depletion of EV batteries, thereby promoting the consumption of fossil fuels.

### Managerial implications

Based on our empirical findings, we offer implications for the decisions of stakeholders like policymakers, EV manufacturers, and EVCP constructors.

For policymakers, first, given that substantial EV purchase subsidies may occupy a large fraction of governmental budgets and consequently hinder the construction of public EVCPs, such subsidies need to be fully monitored and stringently regulated. Second, city-level characteristics should also be accounted for EVCP construction, especially under lower temperatures and during winter, as our results show that constructing more public EVCPs in low temperature periods or regions could notably propel the sales volumes of EVs. Third, as public EVCPs are continuously expanding, policymakers should also encourage the research and development of EV charging technologies to overcome the extant technical limitations such as increasingly slowed charging speed due to the degradation of battery performance.^83^

For EV manufacturers, first, given the significant bidirectional relationship between EV sales and EVCPs, maximum endurance mileage and fast charging capabilities need to be largely enhanced. Second, city-level characteristics can be considered in the launch of new EVs, given that such attributes could significantly impact the relationship between EV sales and EVCPs. For instance, the marketing of EVs can be more frequently rendered in less polluted cities, and marketing strategies can be adopted for residents’ purchasing power and EV purchase subsidies.^35^ As such subsidies are gradually phased out,^84^ EV manufacturers should focus more on whether EV attributes meet market demands.^74^ Third, industrial collaboration can be strengthened to construct and share public EVCPs, with the objective of meeting the ever-increasing charging requirements of EVs, in particular BEVs.

For EVCP constructors, first, since a significant bidirectional relationship between EV sales and EVCPs exists, the fluctuations of EVs sales and local conditions need to be reconsidered during the construction of public EVCPs. Second, it is advisable to expand the construction of public EVCPs in low temperature regions, as lower temperatures are proved to exhibit a much stronger stimulating effect of public EVCPs on EV sales. Third, during the design, planning and construction of public EVCPs, the discrepancies in the charging demands of EVs with different powertrains should be considered.^85^

### Limitations of the study

Admittedly, our research still suffers three major limitations. First, due to limited data availability, we only consider EVs’ purchase subsidies. Additionally, we must admit that the cities with EV sales data are much more than those public EVCP data. To obtain a balanced dataset, we eliminate the cities without EVCPs data, following the practice of Zheng et al. (2022).^37^ Second, several related factors like the technical performances of EVs are neglected due to data availability. There are three major limitations such as the limited data availability of the sales data on specific EV brands and models; the measurements of technical features cannot be standardized, e.g., charging speed, connectivity level, driving feelings, and comfort; Certain technical performance indicators like the levels of autonomous driving cannot be quantitatively measured. Third, we have not included residents’ beliefs and other personal attributes, such as the brand loyalty and commuting frequency. These limitations would provide potential arenas for future research.

### Conclusion

The present empirical evidence for the bidirectional relationship is limited, as most studies are based on mathematical modeling.^9^^,^^10^ Based on a unique panel data of 95 Chinese cities from December 2018 to December 2022, in this article we empirically examine the bidirectional relationship between EV sales and EVCPs, as well as the underlying factors influencing this bidirectional relationship. We find that the bidirectional relationship between EV sales and EVCPs is both significant and positive. The change in EV sales is mainly self-driven and the impact gradually declines with time. The decisions of EVCP investors are highly responsive to the EV market, suggested by the short-term promoting impact of EV sales on EVCPs. Also, our examination identifies the influences of four city-level characteristics on this this bidirectional relationship. Lower temperature increases the stimulating effect of public EVCPs on EV sales due to deteriorated battery performance. Higher air pollution inhibits the positive impact of EV sales on the accessibility of the public EVCPs. High housing price weakens the promotion effect of EVCPs on EV sales due to high construction costs increasing EV usage costs and consumers’ attentions on EVs’ attributes. Substantial EV purchase subsidies also weaken the bidirectional relationship between EV sales and EVCPs. Additionally, we show that EVCPs have a more significant promotional effect on BEVs than on PHEVs, attributing their differentiated powertrains. We also offer implications based on our findings.

## Resource availability

### Lead contact

Further information and requests for resources and reagents should be directed to and will be fulfilled by the lead contact, Fu Gu (gufu@zju.edu.cn).

### Materials availability

This manuscript did not employ any physical material. It is a pure data analysis work.

### Data and code availability

• The EV sales and EVCPs data used in this manuscript has a legal host. Interested readers can contact the hosting institution for the data. Other datasets are available with access to the listed websites in the key resources table.
• The codes used to obtain the results in this study can be made available to interested readers upon reasonable request from the corresponding authors.
• Further clarification and information on the manuscript and data should be addressed to the corresponding authors with the coauthors in copy.

## Acknowledgments

This study is supported by the 10.13039/501100001809National Natural Science Foundation of China (No: 72334007).

## Author contributions

Conceptualization, C.Q., G.F., G.J., L.S., and X.B.; Methodology, X.B.; Data collection, G.J. and X.B.; Writing – original draft, G.F. and X.B.; Writing – review and editing, G.F., G.J., L.S., and Z.X.; Visualization, X.B. All authors have accepted responsibility for the entire content of this manuscript and approved its submission.

## Declaration of interests

The authors declare no competing financial interests.

## STAR★Methods

### Key resources table

**Table:** 

REAGENT or RESOURCE	SOURCE	IDENTIFIER
**Deposited data**		

EV sales	China Banking and Insurance Regulatory Commission (CBIRC)	https://www.cbirc.gov.cn/cn/view/pages/index/index.html
The stock of EVCPs	China Electric Vehicle Charging Infrastructure Promotion Alliance	http://en.caam.org.cn:9527/
Temperature	ERA5-Land Dataset released by European Union and European Centre for Medium-Range Weather Forecasts (ECMWF)	https://cds.climate.copernicus.eu/cdsapp#!/dataset/reanalysis-era5-land-monthly-means?tab=overview
Air pollution	PM2.5 history data	https://www.aqistudy.cn/historydata/
Housing price	Anjuke’s official platform	https://haikou.anjuke.com/?pi=PZ-baidu-pc-all-biaoti
EV purchasing subsidy	The websites of related governments, including The Development and Reform Commission, the Department of Industry and Information Technology, the Department of Finance, the Department of Transport, and the Municipal Government Office.	https://www.ndrc.gov.cn/ https://www.miit.gov.cn/ https://www.mof.gov.cn/index.htm https://www.mot.gov.cn/
Education level	Statistical yearbooks of each province and China population and employment statistical yearbook	N/A
GDP per capita	Statistical yearbooks of each province	N/A
EV enterprises stocks’ trading volume	The financial terminal named Choice of EastMoney; TUSHARE Pro API	https://www.eastmoney.com/ https://tushare.pro/document/2
Purchasing restriction	Official website of each city	N/A
Commercial land use	Statistical yearbook of Chinese urban construction	N/A
Gasoline price	The TUANYOU platform	www.tuanyou.net/youjia/

**Software and algorithms**		

Stata	StataCorp LLC	https://www.stata.com/
Python	Python Software Foundation	http://www.python.org
ArcGIS	Environmental Systems Research Institute	https://www.esri.com/en-us/arcgis/products/index
R	R Core Team	https://cran.r-project.org/
EViews	IHS Global Inc	https://www.eviews.com/home.html
Visual Studio 2021	Microsoft	https://visualstudio.microsoft.com/zh-hans/

### Method details

#### Evaluation of bidirectional relationship using panel vector autoregression

To quantitatively estimate the relationship between variables, we apply the panel VAR approach. This model is proposed by Sims (1980)^86^ and is adapted to analyze variables with strong correlations and bidirectional interactions.^87^ Additionally, the panel VAR has widely been introduced to analyze the bidirectional relationship.^88^^,^^89^

##### Panel stability test

To ensure accurate model estimation and avoid the ‘pseudo regression’ issue, we use two-panel unit root tests, i.e., Levin-Lin-Chu (LLC)^90^ and Phillips-Perron (PP),^91^ to examine whether all variables are stationary; these tests are widely used in previous studies.^27^^,^^92^ If the panel data is found to be unstable, we then determine whether a long-run equilibrium relationship exists between the original variables using cointegration tests^24^^,^^89^ such as the Kao test, the Pedroni test, and the Westerlund test.

##### Model’s lag order selection

To improve model fitting and to prevent loss of degrees of freedom, it is important to select the suitable lag order of variables. Theoretically, too little or too long a lag order compromises model fitting.^24^^,^^27^ This study uses Akaike Information Criterion (AIC), Bayesian Information Criterion (BIC), and Hannan-Quinn Information Criterion (HQIC) to determine the suitable lag order.

##### Granger causality relationship examination

The Granger causality test is adapted to examine whether a change in one time series can lead to subsequent change in another time series.^93^ It can indicate one-way or bidirectional causality. Although the Granger causality does not represent real causality, the statistical significance is not affected and thus is widely applied to explore two-way causality.^88^^,^^89^^,^^94^

##### Quantitative evaluation using generalized method of moments

Quantitative relationships are clarified using GMM estimation, which can be used to address endogeneity issues.^95^ This paper uses GMM to explore the interaction dynamics between EV sales and other variables. The following model is set up:

(Equation 1) $$Y_{it}=\sum_{j=1}^{n}\beta_{j}Y_{i,t-j}+\lambda E_{i,t}+\alpha_{i}+\gamma_{t}+\varepsilon_{i,t}$$

where *i* and *t* denote city and month, respectively. *Y*_*i,t*_ *=* $\left\{\overset{1}{EV\;sales_{it}},\overset{2}{EVCPs_{it}}\right\}$, which *Y*_*i,t*_ is a vector containing two variables. ***E***_***i,t***_ is a vector including exogenous covariates. $\beta_{j}$ and *λ* are the parameters to be estimated. $\alpha_{i}$ is designed to capture the individual fixed effect. The time effect term $\gamma_{t}$ is applied to capture the time trend characteristics. Helmert process, proposed by Arellano and Bover (1995), is applied to eliminate the individual and time effect.^28^ $\varepsilon_{it}$ denotes a random error. In this model, the lagging *Y*_*i,t*_ can be used as instruments and $\beta_{j}$ and *λ* are estimated through GMM.

##### Long-short term relationships exploration

The impulse response function reveals long-short-term relationships between variables by describing the influence of a standard deviation shock on other variables’ current and future states. The Monte Carlo method simulates 1,000 times for each variable with a 10-month impulse response. Variance decomposition is adapted to deal with dynamic stochastic systems, which is a random value process.^24^ This method calculates one variable’s contribution to the standard deviation of the response variable in different periods^24^

#### Moderating impact exploration using two-stage least squares

In this paper, due to the endogeneity exists in two side of EV market, i.e., EV sales and the public EVCPs, might lead to biased regression results. Therefore, 2SLS model by incorporating instrumental variables, are used to overcome the issue.^24^ The regression model of EV sales in the first stage is set as Equation 2, reference to,^8^ we further improve the instrumental variables as commercial land use, which is directly related to the special distribution but relatively exogenous to the EV sales. Several studies have studied the close correlation between the public EVCPs and commercial land use.^8^^,^^40^ A ancillary services would attract the consumers when they need to charge such as vehicle repair and super markets.^9^ The estimated value of EVCPs in the first stage could be introduced in Equation 3 as exogenous part of the endogenous variable.

(Equation 2) $${EVCPs}_{i,t}=\tau +\omega {CL}_{i,t}+X_{i,t}\times \phi +\alpha_{i}+\gamma_{t}+\varepsilon_{i,t}$$

(Equation 3) $${EV\;sales}_{i,t}=\rho +{EVCPs}_{i,t}+X_{i,t}\times \phi +\alpha_{i}+\gamma_{t}+u_{i,t}$$

The regression model of EVCPs in the first stage is shown in Equation 4. Considering the purchasing behavior of consumers could be largely influenced by use costs.^24^ Gasoline price is an effective instrument, which is strongly correlated with EV sales and not directly associated with EVCPs.^8^ The estimated value of EV sales in the first stage could be introduced in Equation 5 as exogenous part of the endogenous variable.

(Equation 4) $${EV\;sales}_{i,t}=\tau +\omega {Gas}_{i,t}+X_{i,t}\times \phi +\alpha_{i}+\gamma_{t}+\varepsilon_{i,t}$$

(Equation 5) $${EVCPs}_{i,t}=\rho +{EV\;sales}_{i,t}+X_{i,t}\times \phi +\alpha_{i}+\gamma_{t}+u_{i,t}$$

#### Bidirectional relationship in terms of electric vehicle types

The roles of BEVs and PHEVs might be differentiated due to their distinct powertrains.^40^ Consumer preferences for these vehicles may be influenced by various factors such as geographic locations, personal beliefs, and the availability of support infrastructures, leading to regional differentiated sales volumes of EVs. With this regard, conducting a Granger causality test to analyze the heterogeneity of EVCPs across BEVs and PHEVs would provide valuable insights into the complicated relationship between EVCPs and EV types.

#### Fully modified ordinary least squares and dynamic ordinary least squares

To explore the robustness of the impact of city-level characteristics on the bidirectional relationship between EV sales and EVCPs, we use FMOLS and DOLS estimators. These methods have been extensively employed to test the robustness of results.^94^^,^^96^^,^^97^ The FMOLS, proposed by Phillips and Hansen (1990),^98^ adopts a semi-parametric approach to estimate the long-run parameters.^99^^,^^100^ The FMOLS estimator is asymptotically unbiased and fully efficient normal asymptotic, allowing for standard Wald tests using asymptotic chi-squared statistical inference.^101^ FMOLS first modifies the variables and estimates directly to eliminate the existing nuisance parameters.^99^

Consider the time-series vector process ${\left(y_{t},x_{t}^{\prime }\right)}^{\prime }$ with cointegrating relationships

(Equation 6) $$y_{t}=x_{t}^{\prime }\beta +{D_{1t}}^{\prime }\gamma_{1}+u_{1t}$$

where *D*_*1t*_ and *D*_*2t*_ are deterministic trend regressors. *D*_*1t*_ enters into both the cointegration Equation 6 and the regressors Equation 7 while *D*_*2t*_ only into the regressors Equation 7. The *n* stochastic regressors *x*_*t*_ are governed by the system of equations:

(Equation 7) $$x_{t}=\Gamma_{21}^{\prime }D_{1t}+\Gamma_{22}^{\prime }D_{2t}+\varepsilon_{2t}$$

(Equation 8) $$\Delta \varepsilon_{2t}=u_{2t}$$

where ${\hat{u}}_{1t}$ is the residuals obtained after estimating Equation 6. ${\hat{u}}_{2t}$ are the differenced residuals of regressor equations which could be obtained as ${\hat{u}}_{2t}=\Delta {\hat{\varepsilon }}_{2t}$, or also could be obtained from the difference regressions:

(Equation 9) $$\Delta x_{t}={\hat{\Gamma }}_{21}^{\prime }\Delta D_{1t}+{\hat{\Gamma }}_{22}^{\prime }\Delta D_{2t}+{\hat{u}}_{2t}$$

Given the long-run covariance matrices $\hat{\Omega }$ and $\hat{\Lambda }$*,* which calculated using the residuals ${\hat{u}}_{t}={\left({\hat{u}}_{1t},{\hat{u}}_{2t}\right)}^{\prime }$, we can define the modified data as following Equation 10.

(Equation 10) $$y_{t}^{+}=y_{t}-{\hat{\omega }}_{12}{\hat{\Omega }}_{22}^{-1}{\hat{u}}_{2t}$$

and an estimated bias-correction terms

(Equation 11) $${\hat{\lambda }}_{12}^{+}={\hat{\lambda }}_{12}-{\hat{\omega }}_{12}{\hat{\Omega }}_{22}^{-1}{\hat{\Lambda }}_{22}$$

The FMOLS estimator is then given by

(Equation 12) $$\hat{\theta }=\left[\begin{array}{c} \hat{\beta }\\ {\hat{\gamma }}_{1} \end{array}\right]=\left[\sum_{t=1}^{T}z_{t}{z^{\prime }}_{t}\right]\;\left[\sum_{t=1}^{T}z_{t}{z^{\prime }}_{t}y_{t}^{+}-\begin{array}{c} T\left(\begin{array}{c} {{\hat{\lambda }}_{12}^{+}}^{\prime }\\ 0 \end{array}\right) \end{array}\right]$$

It worth noting that $y_{t}^{+}$ and ${\hat{\lambda }}_{12}^{+}$ are the correction terms for endogeneity and serial correlation.^99^ This method provides consistent parameter estimations even in small samples, while resolving challenges related to endogeneity, serial correlation, omitted variable bias, and measurement errors. Additionally, it accounts for the heterogeneity in long-term parameters.^100^^,^^102^^,^^103^

The DOLS estimators, proposed by Stock & Watson (1993),^104^ adds the lead and lag of $\Delta x_{t}$ to soak up the long-run correlation between $u_{1t}$ and $u_{2t}$*.* The estimators of DOLS methodology can be obtained through least-squares estimation. These estimators remain unbiased and exhibit asymptotic efficiency, even in the presence of endogeneity issues.^99^

(Equation 13) $$y_{t}=x_{t}^{\prime }\beta +D_{1t}^{\prime }\gamma_{1}+\sum_{j=-q}^{r}\Delta x_{t+j}^{\prime }\delta +v_{1t}$$

where β in Equation 13 is the long-run elasticity. The term δ are the coefficients of leads and lags differences of I (1) regressors. These coefficients serve to adjust for possible endogeneity, autocorrelation, and non-normal residuals.^99^^,^^105^

#### Data description

##### Variables and data sources

Our unique dataset covers 95 major cities, see Table S1 in China and spans from January 2018 to December 2022. We include a comprehensive list of endogenous and exogenous factors, and their detailed information is given in Table 9. To demonstrate the uniqueness of our dataset, we summarize the research focuses, the selected variables, coverage, and periods of their datasets in Table S3.

The EV sales and EVCPs dataset of this paper are collected based on two considerations, i.e., data availability and data representativeness. For data availability, our dataset demonstrates the advantages in terms of data volume, data accuracy (i.e., without the use of any proxy variables), data fineness, and data uniqueness (i.e., being a non-scrapable dataset), which are specified as follows. First, concerning data volume, the observations of datasets in the most of studies are less than 4,300 and contain lots of missing values.^8^^,^^17^^,^^46^^,^^47^ Our dataset includes 5,700 observations, more than those of the datasets used in the existing studies. The comparison between the datasets of the existing literature and this manuscript is shown in Table S9. Second, concerning data accuracy, Gao et al. (2023) admitted that the number of charging stations at the city level is proxied by public financial expenditure.^36^ This would certainly compromise the validity of their empirical results, since public financial expenditure is not equivalent to the stock of EVCPs. For instance, there are huge differences in the procurement and installation costs of various charging stations and land acquisition expenses.^124^ Furthermore, private companies can also construct the public EVCPs.^125^ Our study uses a unique dataset of the public EVCPs, free from using any proxy variables. Third, concerning data fineness, generally, the datasets in the existing studies mostly include yearly data.^5^^,^^17^^,^^47^^,^^126^ Our study employs a unique monthly city-level dataset, which is much more comprehensive and detailed than the dataset used in any previous publication. In our manuscript, we also explain the representativeness of our unique dataset in Table S10 and Figure S1. Fourth, concerning data uniqueness, EV sales data are sourced from the China Banking and Insurance Regulatory Commission and, public EVCP data are sourced from China Electric Vehicle Charging Infrastructure Promotion Alliance (EVCIPA), which is not publicly available. Additionally, in the original dataset, we must admit that the cities with EV sales data are much more than those with the data of public EVCPs. To obtain a balanced dataset, we eliminate the cities that have no EVCPs data and include at least one representative city of each province, following Shang et al. (2024).

We explain the representativeness of our dataset from data volume and distribution. First, for data volume, the data from the included 95 cities covers over 80% of the national EV sales figure and 90% of the national EVCPs, see Panel 1 of Table S10. During 2018 to 2022, the total EV sales of the selected 95 cities and the national figure are 8,913,362 and 11,026,247, respectively. Our EV data accounts for 80.84% of the national EV sales figure. Figure S1A shows EV sales data from the selected 95 cities and national EV sales data. During 2018 to 2022, the total stock of public EVCPs in China is 1,571,626, 1,417,692 of which are built in the selected 95 cities. Our EVCP data accounts for 90.21% of the national figure, demonstrating that our dataset is sufficiently representative, see Panel 2 of Table S10. Second, for data distribution, Figures S1C and S1D illustrate that most of cities with fewer sales are not included. In other words, the EV sales volumes from the selected 95 cities reflect the trend and characteristics of the national EV sales. Figure S1E illustrates the distribution of the nation-wide public EVCPs in China by the end of 2022. Compared to the other cities, the included cities have a relative greater number of EVCPs, again proving the representativeness of our unique dataset, see Figure S1F.

For temperature, Equation 2 shows the composition of the chemical reaction rate (*K*). *A* represents the molecule collision rate, *E*_*A*_ denotes the minimum energy necessary to start the chemical reactions, *R* is the universal gas constant, and *T* represents the temperature in Kelvin.^127^

(Equation 14) $$K=Ae^{-E_{A}/RT}$$

For purchase subsidies, we introduce this variable for the following three reasons. First, from the effect of the phase-out and withdrawal of purchase subsidies are considered in this paper. Although all local purchase subsidies in China were canceled as of June 2019, the national purchase subsidies always exist until the end of our sample period (2022). Between 2017-2019, China reduced EV purchase subsidies by 20%-40% compared to 2016.^128^ Instead of eliminating them, the government planned gradual cuts of 10%, 20%, and 30% per year, ending subsidies in 2022.^129^ Second, from variable perspective, purchase subsidies applied in this paper are a continuous variable, cities that have no subsidies could be noted as zero. We investigate and refer to the policy documents of all 95 cities, mainly from each city government’s official website, to determine whether the incentive policies studied in this paper were under implementation from January 1, 2018, to December 31, 2022. This variable reflects the exact subsidy for each city instead of any proxy variable or dummies. Third, from the data processing perspective, we avoid the loss in the information of policy intensity. Continuous true values in purchase subsidies are relatively convenient to characterize city-level differences and avert the loss of information.^37^ Shang et al. (2024) used EV purchase subsidies data from 2016 to 2019 and prove purchase subsidies could promote EV sales but with a diminishing marginal effect. The continuous values of purchase subsidies could also reflect two aspects of information including center and local governments. Several cities have subsidies in this period although not all of them. This is because the government policies may vary across locations. Our dataset also includes the before and after cancellation of purchase subsidies. Similar to our study, Zhang et al. (2024) also included two stages of before and after subsidy cancellation.^130^ Therefore, neglecting this factor directly may have issues with omitted variables, which also contribute to that the intensity of purchase subsidies has been demonstrated that plays an important role in accelerating EV adoption.^37^^,^^46^

We present the Pearson correlation between all the included variables in Figure 5, which indicates the presence of a high positive correlation between EV sales and EVCPs exceeding 0.7. From Figure 5, EVCPs show a significant positive correlation with temperature and a significant negative correlation with air pollution. In sum, Figure 5 demonstrates the existence of certain correlations between the variables, confirming the validity of variable selection.

##### Descriptive statistics

The descriptive statistics of the variables’ values in our unique dataset are summarized in Table 10. The mean value of monthly EV sales volumes is 1,563.748, while its median value is only 405.5. This reveals that a minority of the included cities contribute the most to EV sales. BEV sales are about 4 times as large as PHEV sales. In terms of standard deviation, the distribution of EV sales is more volatile than EVCPs. There are considerable disparities in the maximal and minimal values of EV sales (including the sales of both BEVs and PHEVs) and EVCPs, indicating the existence of substantial variations among the included cities. The mean value of purchase subsidies is 4.016, while the maximum is 14.400, suggesting that city-level purchase subsidies could be highly differentiated. The mean value of air pollution is 60.056 and the maximum is 206.927, suggesting that the overall pollution level in most of the included cities is rather low. The skewness values of all the exogenous variables are less than or merely more than 1, therefore the values of these variables are approximate normally distributed.

### Quantification and statistical analysis

All statistical analyses were performed in Stata, Python, R, ArcGIS, and EViews.

## Supplemental information

Document S1. Figure S1 and Tables S1, S2, and S4–S10

Table S3. Former research, related to STAR methods
