# Supplementary material for: Unveiling the bidirectional link between electric vehicle sales and charging infrastructure: Evidence from 95 cities in China
Source: iScience. 2024 Oct 24;27(11):111245. doi: 10.1016/j.isci.2024.111245 (PMC11574808; doi:10.1016/j.isci.2024.111245)
Supplement: Document S1. Figure S1 and Tables S1, S2, and S4–S10 [file mmc1.pdf]

iScience, Volume *27*

Supplemental information

Unveiling the bidirectional link between electric vehicle sales and charging infrastructure: Evidence from 95 cities in China

Jianfeng Guo, Binbin Xu, Qi Cao, Siyao Liu, Fu Gu, and Xuemei Zhang

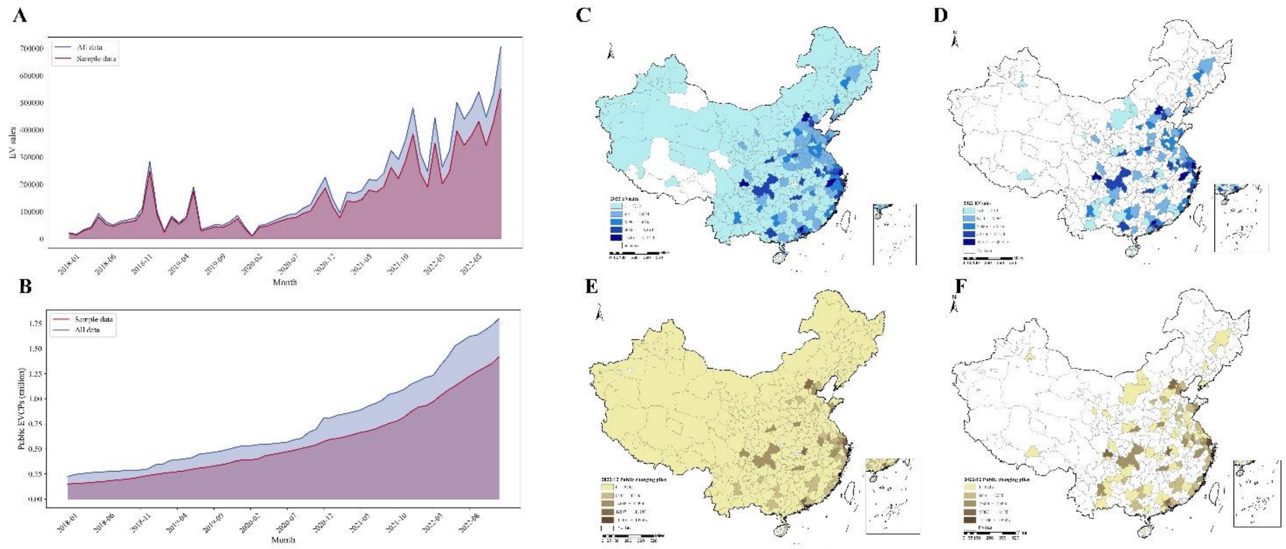

Figure S1. Comparison of selected cities' EV sales and EVCPs with all cities, related to data description

(A) The evolution of EV sales from 2018 to 2022. The blue part denotes the EV sales data at the national level, and the red part denotes the sample data in our dataset. (B) The evolution of the public EVCPs from 2018 to 2022. The blue part denotes the national EVCP data, and the red part denotes the sample data. It worth noting that the aggregated EVCP data is acquired from Pilot Research Report (2023), and gaps persist due to statistical caliber. (C) The distribution of the regional EV sales from 2018 to 2022. The blue color indicates the EV sales volumes, and the higher color intensity corresponds to the greater quantity. (D) The EV sales distribution within the studied cities from 2018 to 2022. (E) The national public EVCPs distribution by the end of 2022. The yellow color denotes the stock of the EVCPs in such regions, and the higher color intensity corresponds to the greater quantity. (F) The public EVCPs distribution within the studied cites by the end of 2022.

Table S1. Included cities in this paper, related to the variables and data sources in the STAR methods

Province	City
Anhui	Fuyang, Wuhu, Chuzhou, Hefei, Bozhou
Beijing	Beijing
Fujian	Longyan, Fuzhou, Zhangzhou, Quanzhou, Xiamen
Gansu	Lanzhou
Guangdong	Dongguan, Zhaoqing, Zhuhai, Qingyuan, Shenzhen, Jiangmen, Huizhou, Guangzhou, Foshan, Zhongshan
Guangxi	Liuzhou, Yulin, Hechi, Guilin, Nanning
Guizhou	Bijie, Zunyi, Guiyang, Sanya, Haikou, Danzhou, Handan, Shijiazhuang, Langfang, Tangshan, Baoding
Henan	Xinxiang, Zhengzhou, Xuchang, Luoyang, Anyang
Heilongjiang	Harbin
Hubei	Yichang, Wuhan, Huanggang, Jingzhou
Hunan	Changsha
Jilin	Changchun
Jiangsu	Suzhou, Taizhou, Yancheng, Wuxi, Yangzhou, Xuzhou, Changzhou, Suqian, Nantong, Nanjing
Jiangxi	Ganzhou, Yichun, Nanchang
Liaoning	Shenyang, Dalian
Inner Mongolia	Ordos, Hohhot, Baotou
Ningxia	Yinchuan
Qinghai	Xining
Shandong	Jining, Jinan, Linyi, Qingdao, Weifang
Shanxi	Taiyuan
Shaanxi	Xi'an, Baoji, Xianyang
Shanghai	Shanghai
Sichuan	Mianyang, Meishan, Chengdu
Tianjin	Tianjin
Tibet	Lhasa
Sinkiang	Urumqi
Yunnan	Kunming, Honghe, Qujing
Zhejiang	Wenzhou, Hangzhou, Ningbo, Jiaxing, Taizhou
Chongqing	Chongqing

Table S2. Abbreviation's explanation, related to introduction

Abbreviations	Full term
EV	Electric vehicle
BEV	Battery electric vehicle
PHEV	Plug-in hybrid electric vehicle
EVCP	Electric vehicle charging piles
AQI	Air quality index
PVAR	Panel vector autoregression
DID	Difference-in-differences
PSM	Propensity score matching
TSA	Text sentiment analysis
PCSE	Panel-corrected standard error multivariate regression
DIF-GMM	The difference generalized method of moments estimator
SYS-GMM	The system generalized method of moments estimator
DCM	Discrete choice model
CS-ARDL	Cross-sectional augmented autoregressive distributed lag model
GLM	Generalized Linear Model
FMOLS	Fully modified ordinary least squares
DOLS	Dynamic ordinary least squares
2SLS	Two-stage least squares

Table S4. Results of Data stability test, related to "The bidirectional relationship between EV sales and EVCPs" in the results

**Panel 1: Results of panel unit root tests**					
**Variable**	**Level**		**1st difference**		**Order of integration**
	**Intercept**	**Intercept & trend**	**Intercept**	**Intercept & trend**	
**Levin-Lin-Chu (LLC) test**					
EV sales	-21.6790***	-40.4490***	-104.2520***	-105.2170***	I (1)
EVCPs	72.6210	19.2290	-36.8450***	-58.9760***	I (1)
**Phillips-Perron (PP) test**					
EV sales	700.3765***	1174.5840***	6706.6089***	6432.7718***	I (1)
EVCPs	0.2913	13.9879	1917.8609***	2773.5405***	I (1)

**Panel 2: Co-integration test results**			
**Test method**	**Statistics**	**Statistics value**	**p-value**
Kao test	*Modified Dickey-Fuller t*	-24.4474	0
	Dickey-Fuller t	-14.3813	0
	Augmented Dickey-Fuller t	-4.1972	0
	Unadjusted modified Dickey-Fuller t	-51.0824	0
	Unadjusted Dickey-Fuller t	-20.0821	0
Pedroni test	*Modified Phillips-Perron t*	-47.1090	0
	Phillips-Perron t	-36.4065	0
	Augmented Dickey-Fuller t	-36.7113	0
Westerlund test	*Variance ratio*	-10.0412	0

**Panel 3: Selection of the lag order**			
**lag**	**AIC**	**BIC**	**HQIC**
1	32.8369*	33.0698*	32.9181*
2	35.0449	35.2861	35.1291
3	34.5243	34.7741	34.6116

*, **, and *** in Panel 1 and 2 indicate statistical significance at the 10%, 5%, and 1% level, respectively. * in Panel 3 indicates lag order selected by the criterion.

Table S5. Variance decomposition of variables, related to Figure 4

Response variables	Forecast period	*EV sales*	*EVCPs*
EV sales	1	1	0
EV sales	2	0.9997	0.0003
EV sales	3	0.9991	0.0009
EV sales	4	0.9985	0.0015
EV sales	5	0.9979	0.0021
EV sales	6	0.9972	0.0028
EV sales	7	0.9964	0.0036
EV sales	8	0.9955	0.0045
EV sales	9	0.9945	0.0055
EV sales	10	0.9935	0.0065
EVCPs	1	0.2703	0.7297
EVCPs	2	0.1520	0.8480
EVCPs	3	0.1038	0.8962
EVCPs	4	0.0780	0.9220
EVCPs	5	0.0619	0.9381
EVCPs	6	0.0509	0.9491
EVCPs	7	0.0430	0.9570
EVCPs	8	0.0370	0.9630
EVCPs	9	0.0323	0.9677
EVCPs	10	0.0286	0.9714

Table S6. Outcomes of selecting order criteria for panel VAR, related to "Electric vehicle types" in the results

lag	AIC	BIC	HQIC
**Panel 1:** ***BEV EVCPs***			
1	30.1893*	30.4221*	30.2705*
2	30.7446	30.9859	30.8288
3	31.0515	31.3013	31.1388
**Panel 2:** ***PHEV EVCPs***			
1	32.8369*	33.0698*	32.9181*
2	35.0449	35.2861	35.1291
3	34.5243	34.7741	34.6116

* indicates lag order selected by the criterion.

Table S7. Definitions, data sources and justifications of the substitute variables, related to robustness checks

Original variables	Substitute variables	Variable definition and data source	Justification
T	*MaxT*	The monthly average daily maximum temperature in each included city. The data is acquired from National Center for Environmental Information (NCEI) (www.ncei.noaa.gov/data/global-summary-of-the-day/archive/).	This variable is used to measure the changes of temperature [S13].
AP	*CO*	Carbon monoxide emission in each included city. The data is collected from PM2.5 historic data (www.aqistudy.cn/historydata/).	This variable a common indicator to measure air pollution [S14].
SHP	*NBHP*	Newly-built housing price in each included city. The data is acquired from Anjuke's official platform (hechi.anjuke.com/sale/?from=HomePage_TopBar)	This variable reflects the level of housing prices [S15], though which are regulated by governments [S16,17].
PS	*2PS*	200 mileage standard subsidies in each included city. The data is gathered from the websites of related governments, including The Development and Reform Commission, the Department of Industry and Information Technology, the Department of Finance, the Department of Transport, and the Municipal Government Office.	Referring to Chen *et al*. (2016), this variable serves as alternative measurement corresponding to the upper limit of the monthly EV subsidy [S1].

Table S8. Outcomes of the robustness checks, related to the robustness checks.

Panel 1: Robustness check with 2SLS.

**Variables**	***EV sales***	***EVCPs***
	**2SLS**	**2SLS**
	**(1)**	**(2)**
EVCPs	0.181***	
	(8.540)	
EV sales		3.822**
		(2.070)
T	-4.545	11.317
	(-1.110)	(0.504)
AP	-1.399	4.142
	(-0.855)	(0.533)
SHP	0.158***	-0.139
	(4.390)	(-0.173)
PS	74.426**	-457.992***
	(3.283)	(-4.858)
EL	720.299**	-55.823
	(2.528)	(-0.013)
GDP	0.080***	-0.290
	(3.647)	(-1.546)
MA	-3.057***	9.192
	(-4.913)	(1.056)
PR	-205.580	901.476
	(-0.445)	(0.470)
First-stage IV	237.198***	117.211**
	(12.96)	(1.97)
Wald chi^2	8176.280	6121.660

Panel 2: Robustness check with moderating impact of *temperature*.

**Variables**	***EV sales***			***EVCPs***		
	**2SLS**	**FMOLS**	**DOLS**	**2SLS**	**FMOLS**	**DOLS**
	**(1)**	**(2)**	**(3)**	**(4)**	**(5)**	**(6)**
EVCPs	0.307***	0.471***	0.127**			
	(3.786)	(27.445)	(2.847)			
EVCPs × T		-0.003***	0.005			
		(-7.058)	(3.565)			
EVCPs × maxT	-0.005*					
	(-2.104)					
EV sales				0.696	0.781***	0.902***
				(0.754)	（14.007）	(6.265)
EV sales × T					0.014***	0.026***
					(5.584)	(4.883)
EV sales × maxT				0.041		
				(1.363)		
First-stage IV	63.794***			-182.642***		
	(6.700)			(-5.170)		
Wald chi2	8,869.380			12,006.260		

Controls	Y	Y	Y	Y	Y	Y
**Panel 3: Robustness check with moderating impact of *air pollution*.**						
**Variables**	***EV sales***			***EVCPs***		
	**2SLS**	**FMOLS**	**DOLS**	**2SLS**	**FMOLS**	**DOLS**
	**(1)**	**(2)**	**(3)**	**(4)**	**(5)**	**(6)**
EVCPs	0.331*	0.342***	0.321***			
	(2.236)	(16.998)	(7.959)			
EVCPs × AP		0.002***	-0.001			
		(7.039)	(-1.455)			
EVCPs × CO	-0.244			3.405***		
	(-1.185)			(3.337)		
EV sales					1.477***	1.801***
					(22.184)	(13.856)
EV sales × AP					-0.009***	-0.007**
					(-6.257)	(-2.405)
EV sales × CO				-2.545*		
				(-1.964)		
First-stage IV	35.602			163.964***		
	(5.550)			(9.140)		
Wald chi²	9,106.900			11,596.230		
Controls	Y	Y	Y	Y	Y	Y
**Panel 4: Robustness check with moderating impact of *housing price*.**						
**Variables**	***EV sales***			***EVCPs***		
	**2SLS**	**FMOLS**	**DOLS**	**2SLS**	**FMOLS**	**DOLS**
	**(1)**	**(2)**	**(3)**	**(4)**	**(5)**	**(6)**
EVCPs	0.345*	0.985***	-0.266			
	(2.471)	(3.926)	(-0.460)			
EVCPs × SHP		-7.38E-05***	7.59E-05			
		(-2.616)	(1.170)			
EVCPs × NBHP	-3.52E-06					
	(-1.488)					
EV sales				5.978	2.980***	2.163
				(1.072)	(4.381)	(1.642)
EV sales × SHP					-0.0002**	0.0001
					(-2.103)	(0.685)
EV sales × NBHP				-0.0001		
				(-0.676)		
First-stage IV	35.538***			42.809		
	(4.560)			(1.440)		
Wald chi²	9131.200			6152.77		
Controls	Y	Y	Y	Y	Y	Y
**Panel 5: Robustness check with moderating impact of *purchase subsidies*.**						
**Variables**	***EV sales***			***EVCPs***		
	**2SLS**	**FMOLS**	**DOLS**	**2SLS**	**FMOLS**	**DOLS**
	**(1)**	**(2)**	**(3)**	**(4)**	**(5)**	**(6)**
EVCPs	0.178***	0.307***	0.349***			
	(8.682)	(11.854)	(4.756)			

EVCPs × PS		0.087***	0.005			
		(6.734)	(0.152)			
EVCPs × 2PS	0.026***					
	(5.429)					
EV sales				2.443*	2.153***	2.912***
				(1.983)	(46.791)	(34.358)
EV sales × PS					-0.513***	-0.746***
					(-27.805)	(-20.262)
EV sales × 2PS				-0.595***		
				(-3.701)		
First-stage IV	243.299***			121.122**		
	(13.810)			(2.190)		
Wald chi^2	8,354.880			13,149.73		
Controls	Y	Y	Y	Y	Y	Y

Panel 6: The Granger causality test result of *BEV sales* and *Newlybuilt*.

Variables	Test items	chi2	*P*
BEV	*Newlybuilt*	16.525	0
Newlybuit	*PHEV*	23.818	0

Panel 7: The Granger causality test result of *PHEV sales* and *Newlybuilt*.

Variables	Test items	chi2	*P*
PHEV	*Newlybuilt*	0.191	0.662
Newlybuilt	*PHEV*	28.448	0

This table reports the robustness results. the *t* statistics are reported in parentheses. Specifically, for 2SLS method, the *t*-value are reported in the first-stage, and the *z*-values are reported in the second stage. *, **, and *** indicate statistical significance at the 10%, 5%, and 1% level, respectively. To maintain brevity, "*Controls*" is used to denote all other control variables.

Table S9 Data comparison between existing research and this paper, related to data description.

Authors	Data Range	Cities	Time period	Observations	Justification	Data source
Zhao *et al*. (2024)	2010-2019	50 Chinese cities	yearly	500		Energy Conservation and New Energy Vehicle Yearbook 2011–2020
Li *et al*. (2017)	2011-2013	353Metropolitan Statistical Areas (MSAs)	quarterly	4236		IHS Automotive
Zheng *et al*. (2022)	2009-2018	286 Chinese cities	yearly	2380	Due to lack of sufficient the charging piles, only 51 cities have EVCPs data	China Energy Saving and New Energy Vehicle Yearbook
Qiu *et al*. (2019)	Jan. 2014-Aug. 2015	88 Chinese pilot cities	monthly	1760		The traffic management bureau of each city
Ma and Fan (2020)	Feb. 2016-Apr. 2018	20 Chines provinces	monthly	540		China Electric Vehicle Charging Infrastructure Promotion Alliance (EVCIPA)<br>China Automotive Technology and Research Center (CATARC)
Khatua *et al*. (2023)	2011-2020	30 countries	yearly	270		IEA; Global EV Outlook 2020, 2021, & 2022.
Kalthaus and Sun (2021)	2010-2016	31 Chinese provinces	yearly	207		The Yearbook of Energy-Saving and New Energy Vehicles in China,
Zhang *et al*. (2016)	2013-2015	88 Chinese cities	yearly	264		Yearbook of China's Fuel Economy and New Energy Vehicles
Shang *et al*. (2024)	2016-2019	224 Chinese cities	monthly	1758	Only less than 50 cities after eliminating missing value	China Energy Saving and New Energy Vehicle Yearbook and Zhi-Yun database
Our study	2018-2022	95 Chinese cities	monthly	5700	No missing value, 345 Chinese cities (original dataset)	China Banking and Insurance Regulatory Commission (CBIRC)<br>China Electric Vehicle Charging Infrastructure Promotion Alliance

Table S10 Statistics of sample data, related to data description.

**Panel 1: The data statistics of EV sales from 2018 to 2022.**		
	**Data of the whole country**	**Data of selected 95 cities**
Number of cities	349.00	95.00
Mean	31,593.83	93,824.86
Standard deviation	80,924.34	136,132.55
Minimum	1.00	1,516.00
25% quantile	2,381.00	17,293.50
50% quantile	8,151.00	45,038.00
75% quantile	23,313.00	108,761.50
Maximum	833,647.00	833,647.00
Sum	11,026,247	8,913,362.00
**Panel 2: The data statistics of public EVCPs constructed by the end of 2022.**		
	**Data of the whole country**	**Data of selected 95 cities**
Number of cities	344.00	95.00
Mean	4,568.68	14,923.07
Standard deviation	15,096.81	27,304.61
Minimum	0	257.00
25% quantile	379.00	3,111.50
50% quantile	1,110.00	5,379.00
75% quantile	2,662.00	14,037.50
Maximum	207,577.00	207,577.00
Sum	1,571,626	1,417,692

REFERENCES

[S1] Ma, S. C., & Fan, Y. (2020). A deployment model of EV charging piles and its impact on EV promotion. *Energy Policy 146*, 111777. https://doi.org/10.1016/j.enpol.2020.111777.

[S2] Zheng, X. M., Menezes, F., Zheng, X. F., & Wu, C. K. (2022). An empirical assessment of the impact of subsidies on EV adoption in China: A difference-in-differences approach. *Transportation research part A-policy and practice 162*, 121-136. https://doi.org/10.1016/j.tra.2022.05.020.

[S3] Yang, Z., Li, Q., Yan, Y., Shang, W.-L., & Ochieng, W. (2022). Examining influence factors of Chinese electric vehicle market demand based on online reviews under moderating effect of subsidy policy. *Applied Energy 326*, 120019. https://doi.org/10.1016/j.apenergy.2022.120019.

[S4] Khatua, A., Kumar, R. R., & De, S. K. (2023). Institutional enablers of electric vehicle market: Evidence from 30 countries. *TRANSPORTATION RESEARCH PART A-POLICY AND PRACTICE 170*, 103612. https://doi.org/10.1016/j.tra.2023.103612.

[S5] Qiu, Y. Q., Zhou, P., & Sun, H. C. (2019). Assessing the effectiveness of city-level electric vehicle policies in China. *Energy policy 130*, 22-31. https://doi.org/10.1016/j.enpol.2019.03.052.

[S6] Zhang., Ou, X. M., Yan, X. Y., & Zhang, X. L. (2017). Electric Vehicle Market Penetration and Impacts on Energy Consumption and CO₂ Emission in the Future: Beijing Case. *ENERGIES 10*, 228. https://doi.org/10.3390/en10020228.

[S7] Liu, Y. R., Zhao, X. L., Lu, D., & Li, X. M. (2023). Impact of policy incentives on the adoption of electric vehicle in China. *Transportation research part A-policy and practice 176*, 103801. https://doi.org/10.1016/j.tra.2023.103801.

[S8] Wu, S. P., & Yang, Z. (2020). Availability of Public Electric Vehicle Charging Pile and Development of Electric Vehicle: Evidence from China. *Sustainability 12*, 6369. https://doi.org/10.3390/su12166369.

[S9] Illmann, U., & Kluge, J. (2020). Public charging infrastructure and the market diffusion of electric vehicles. *Transportation research part D-transport and environment 86*, 102413. https://doi.org/10.1016/j.trd.2020.102413.

[S10] Azarafshar, R., & Vermeulen, W. N. (2020). Electric vehicle incentive policies in Canadian provinces. *Energy Economics 91*, 104902. https://doi.org/10.1016/j.eneco.2020.104902.

[S11] Haidar, B., & Rojas, M. T. A. (2022). The relationship between public charging infrastructure deployment and other socio-economic factors and electric vehicle adoption in France. *Research in transportation economics 95*, 101208. https://doi.org/10.1016/j.retrec.2022.101208.

[S12] Kalthaus, M., & Sun, J. T. (2021). Determinants of Electric Vehicle Diffusion in China. *Environmental & resource economics 80*, 473-510. https://doi.org/10.1007/s10640-021-00596-4.

[S13] Kim, S., Lyu, T. K., & Park, J. W. (2023). Numerical Study on the Effects of Environmental Temperature of Major Cities in California on the Capacity Fade of Battery Cells in Electric Vehicles. *INTERNATIONAL JOURNAL OF AUTOMOTIVE TECHNOLOGY 24*, 1447-1458. https://doi.org/10.1007/s12239-023-0117-3.

[S14] Wallington, T. J., Anderson, J. E., Dolan, R. H., & Winkler, S. L. (2022). Vehicle Emissions and Urban Air Quality: 60 Years of Progress. *ATMOSPHERE 13*, 650. https://doi.org/10.3390/atmos13050650.

[S15] Straka, M., Carvalho, R., Van der Poel, G., & Buzna, L. (2021). Analysis of Energy Consumption at Slow Charging Infrastructure for Electric Vehicles. *IEEE ACCESS 9*, 53885-53901. https://doi.org/10.1109/ACCESS.2021.3071180.

[S16] Yuan, F., Wu, J. W., Wei, Y. D., & Wang, L. (2018). Policy change, amenity, and spatiotemporal dynamics of housing prices in Nanjing, China. *Land use policy 75*, 225-236. https://doi.org/10.1016/j.landusepol.2018.03.045.

[S17] Li, H., Chen, P. J., & Grant, R. (2021). Built environment, special economic zone, and housing prices in Shenzhen, China. *Applied Geography 129*, 102429. https://doi.org/10.1016/j.apgeog.2021.102429.

[S18] Chen, T. D., Kockelman, K. M., & Hanna, J. P. (2016). Operations of a shared, autonomous, electric vehicle fleet: Implications of vehicle & charging infrastructure decisions. *TRANSPORTATION RESEARCH PART A-POLICY AND PRACTICE 94*, 243-254. https://doi.org/10.1016/j.tra.2016.08.020.

[S19] Zhao, X. L., Zhao, Z. Y., Mao, Y. M., & Li, X. M. (2024). The role of air pollution in electric vehicle adoption: Evidence from China. *Transport Policy 154*, 26-39. https://doi.org/10.1016/j.tranpol.2024.05.022.

[S20] Li, S. J., Tong, L., Xing, J. W., & Zhou, Y. Y. (2017). The Market for Electric Vehicles: Indirect Network Effects and Policy Design. *Journal of the association of environmental and resource economists 4*, 89-133. https://doi.org/10.1086/689702.

[S21] Zhang, G. Q., Xu, Y. M., & Zhang, J. (2016). Consumer-Oriented Policy towards Diffusion of Electric Vehicles: City-Level Evidence from China. *Sustainability 8*, 1343. https://doi.org/10.3390/su8121343.

[S22] Shang, W. L., Zhang, J. J., Wang, K., Yang, H. J., & Ochieng, W. (2024). Can financial subsidy increase electric vehicle (EV) penetration---evidence from a quasi-natural experiment. *Renewable & sustainable energy reviews 190*, 114021. https://doi.org/10.1016/j.rser.2023.114021.

[S23] Pilot Research Report. (2023). *China Charging Alliance: Operation of national electric vehicle charging and replacement infrastructure in 2022*. https://www.xdyanbao.com/doc/hg4uluc7c2?bd_vid=7920583722278925807.
